# Public-private partnerships for seed industry development in developing countries: Lessons from MasAgro maize in Mexico

**DOI:** 10.1371/journal.pone.0328872

**Published:** 2025-08-06

**Authors:** Ciro Domínguez, Chittur S. Srinivasan, Arturo Silva-Hinojosa, Iraís Dámaris López-Becerril, Laura Donnet, Giacomo Zanello, Juan Burgueño

**Affiliations:** 1 School of Agriculture, Policy and Development, University of Reading, Reading, United Kingdom; 2 International Maize and Wheat Improvement Centre (CIMMYT), Texcoco, Mexico; ICAR - Indian Agricultural Research Institute, INDIA

## Abstract

Public-private partnerships (PPPs) are globally recognized for their potential to accelerate genetic improvement and delivery of new high-yielding seed varieties in developing countries. However, despite the strong advocacy for PPPs in crop improvement, there is little empirical evidence about their performance, capacities, and contribution to the development of seed industries and the promotion of competitive seed markets. This paper uses the experience of the MasAgro maize consortium, a PPP in Mexico, to examine crop variety innovation and delivery through PPPs, assess PPPs’ capacities to commercialize public germplasm-based varieties, and derive lessons for the design and implementation of future PPPs. Drawing on a combination of multiple data sources, we examined the PPP’s performance in the generation, dissemination, and commercialization of new maize hybrids. Our examination over the period 2011–2019 shows that the consortium was successful in maintaining a substantial flow of agronomically competitive maize hybrids, which compared favourably with the number of new varieties generated by national and international seed companies and the public sector. The partnership also contributed to refreshing and rejuvenating the variety portfolios of the consortium companies, which appear to have succeeded in bringing MasAgro varieties quickly into the market. However, seed sales achieved by MasAgro hybrids over this period remained small and multinational companies consistently maintained their leadership in the maize seed market. Our analysis shows that PPPs have strong capacities for the development of competitive seed varieties, but they face significant challenges in scaling up the uptake and adoption of these innovations in highly concentrated markets. To succeed in their objective of delivering affordable, high-quality seed on a large scale to smallholder farmers in developing countries, PPPs need to urgently incorporate a commercial and market-oriented perspective along all steps of the plant breeding and dissemination process.

## Section 1: Introduction

Many developing countries like India, Brazil and Mexico have relied mainly on crop variety innovations developed by public sector National Agricultural Research Systems (NARS) to drive productivity growth in agriculture. Crop variety innovations from NARS have also underpinned the development of the commercial seed sector in these countries, especially in the initial phases of agricultural development. Developing country NARS, working in collaboration with International Agricultural Research Centres (IARCs) of the Consultative Group on International Agricultural Research (CGIAR) system, are acknowledged to have made exceptional contributions to plant breeding. Such collaboration led to the development of the semi-dwarf, high-yielding wheat and rice varieties that transformed agriculture during the “Green Revolution” in many developing nations, substantially increased food production, and largely contributed to improving global food security [1,2]. The factors driving the success of the public sector in the development and dissemination of crop varieties in the 1960s and 1970s were, to a large extent, the strong interest of governments and donors in investing in agricultural research, and the free exchange of plant genetic resources in the CGIAR that facilitated collaborations between national and international research systems.

While NARS remained dominant in plant breeding in developing countries till the 1990s, and provided the main links with the international plant breeding research system, the effectiveness and sustainability of the public-sector led innovation system have been called into question by several related developments. These include:

1) the declining trends in public investment in agricultural research in developing countries since the 1990s [3,4];
2) the declining efficacy of NARS in the dissemination of crop varieties, reflected in the limited uptake and adoption of new improved seeds [4];
3) growing private sector competition in plant breeding, generated mainly by the expansion of multinational companies but also by the increasing breeding capacity of domestic firms [5,6].
4) the enforcement of intellectual property rights (IPRs) in developed nations since the 1960s and their extension to developing countries in the 1990s [1,2], restricting free germplasm exchange and plant breeders’ capacities to build on protected innovations [7,8];
5) the emergence of biodiversity legislation that recognized national “sovereignty over biological resources”, which further exacerbated the limitations imposed by IPRs for germplasm access and exchange in the international plant breeding research system [2,9].

In this context, public-private partnerships (PPPs) emerged as an alternative to promote seed sector development [10,11], accelerate variety development [12,13], and their delivery at scale to smallholder farmers in developing countries [14,15]. PPPs are collaborations between public and private entities in which partners share costs, risks, and resources in pursuit of a common goal. Such collaborations can range from bilateral cooperation between governments and a private company to multi-partner and multi-sector alliances and research consortia, including governments, private companies, foundations, development organizations, and NGOs [16–18]. PPPs in plant breeding leverage the public sector’s upstream breeding capacities with private sector strengths for seed production, distribution and marketing. Public sector NARS and CGIAR centres provide strength in crop improvement, access to diverse germplasm collections and evaluation networks. The private sector contributes expertise in modern breeding technologies, research skills, tools, marketing and seed delivery systems [12,15,18].

For over the last three decades, the CGIAR has established PPPs with a wide range of actors, especially for the development and dissemination of high-yielding, stress-tolerant, and biofortified seed varieties (S1 Appendix). For example, the International Maize and Wheat Improvement Centre (CIMMYT), the Kenyan Agricultural Research Institute (KARI), and the Syngenta Foundation launched the Insect-Resistant Maize for Africa (IRMA) initiative in 1999 and worked for over fifteen years to develop and deliver stem borer-resistant maize varieties [19]. Similar initiatives such as the Drought Tolerant Maize for Africa (DTMA) [20], the Stress Tolerant Maize for Africa (STMA) [21], and the Affordable, Accessible Asian Drought Tolerant Maize Project (AAA-DT Maize) [22] developed from 2005 to 2020 more than two hundred drought-tolerant and insect-resistant maize cultivars adapted to smallholder farming in Africa and Asia. Along with the CGIAR, development agencies such as the United States Agency for International Development (USAID); foundations, e.g., the African Agricultural Technology Foundation (AATF), the Syngenta, Bill and Melinda, Rockefeller, and Howard G. Buffett Foundations; as well as national and multinational companies (Bayer, BASF, Corteva Agriscience, Syngenta) have been involved in PPPs since the early 1990s. The USAID, the AATF, the Syngenta, and Bill and Melinda Gates Foundations have advocated PPPs since the late 1990s/early-2000s, mostly in Africa, Asia and Latin America [15,23,24].

Within PPPs arrangements in the CGIAR, public breeding programmes typically provide free-of-charge access to elite breeding materials to domestic small and medium enterprises (SMEs), and in return, SMEs multiply and market CGIAR or NARS crop varieties [25]. These arrangements aim to assign a larger role to domestic companies in seed sector development and encourage emerging plant breeding capacity in the private sector. In many developing countries where smallholder farmers are the main buyers of seeds, innovations developed and delivered by PPPs provide an alternative to varieties offered by multinational companies that usually enjoy substantial price premiums and may be unaffordable to low-income farmers.

### Objectives and outline

For many years, PPPs in seed sector development have attracted and continue to attract investments from developed and developing country governments, CGIAR institutions and donor agencies [15,23,24]. A general common assumption when implementing PPPs is that they can produce and distribute high-quality and affordable seeds at large scale to low-income farmers in remote markets. However, several questions remain to systematically evaluate the role of PPPs and their contribution to large scale dissemination of crop varieties and seed sector development. For example, can SMEs successfully incorporate new seed innovations developed through PPPs into their product portfolios and into the market? Can PPPs accelerate variety development, variety turnover and dissemination at scale of new hybrids? Can PPPs contribute to the development of vigorous local seed industries, capture market shares from global market leaders and promote sufficient competition in the seed industry? and, Can PPPs deliver on broader development goals related to achieve crop productivity increases, seed systems development and smallholder crop production which underpin large public sector investments? To the best of our knowledge, there are no empirical evaluations of seed sector PPPs that address the above questions.

This paper examines the performance of MasAgro maize, a PPP in Mexico involving CIMMYT, the main public breeding NARS, the Mexican government, and over seventy SMEs during 2011–2019. It specifically addresses the following research questions:

1. What was the contribution of the MasAgro maize PPP to the development of new hybrid maize varieties in the Mexican maize seed industry over the period 2011–2019?
2. How did MasAgro maize hybrids compare in terms of yield with hybrids of private companies and the public sector?
3. What was the impact of MasAgro maize on the variety portfolios of participating SMEs and on the maize seed market?

The paper aims to build evidence of the contribution that PPPs can make to seed sector development in developing countries, and derive lessons from the MasAgro maize experience to inform the design and implementation of future PPPs in other developing countries where the private sector plays (or can potentially play) a key role in maize seed multiplication and delivery. The analysis is structured as follows. In the next section, we present our conceptual framework; section 3 presents our data sources and methods; section 4 provides the background of maize production and the maize seed industry in Mexico; section 5 presents the results and section 6 discusses the implications of the findings for the design of PPPs and the development of seed industries through PPPs. Section 7 is the conclusion.

## Section 2: PPPs in plant breeding and maize seed systems

**Fig 1:**
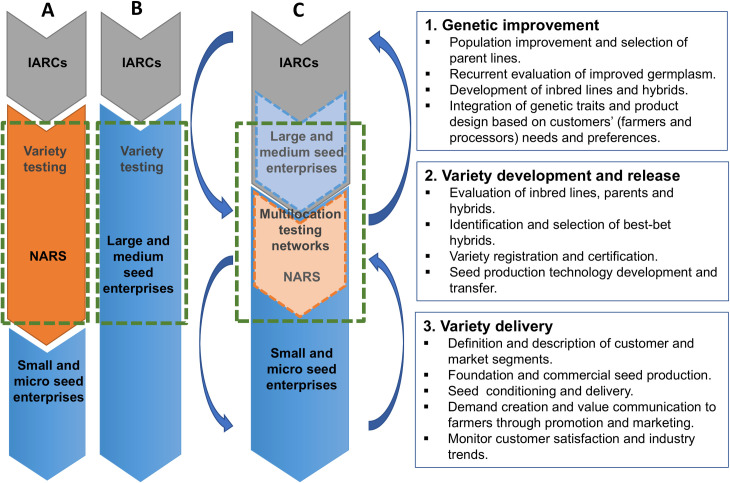
Crop germplasm improvement, evaluation, and delivery through A) the CGIAR-NARS route; B) the private route; and C) public-private partnerships that incorporate a commercial and market perspective along the maize seed value chain. Source: Authors, based on [27–29].

In formal maize seed systems, breeders use germplasm collections stored in gene banks to generate pedigree populations and elite inbred lines for further use by NARS (CGIAR-NARS route) and private companies (private route) for the development of advanced lines and hybrids [1,26]. In the CGIAR-NARS route (Fig 1A), NARS are recipients of CGIAR germplasm for further development and evaluation. NARS are then the main technology and foundation seed providers to private seed companies, which in turn are seed multipliers. NARS and private companies mainly work separately, with private seed firms mostly acting as receivers of technology, knowledge and information. In low-and-middle-income countries, few private seed companies have the capacities to invest in breeding research. Seed companies with plant breeding capacity will usually skip the CGIAR-NARS route, making their own germplasm improvement and variety evaluation by directly accessing genetic resources from the CGIAR or other national/international sources (Fig 1B).

However, the capacity for innovation in individual domestic seed firms may be limited. In public-private partnerships (Fig 1C) public sector entities provide a broad range of elite breeding materials to domestic seed companies to stimulate their collective plant breeding capacity, while exploiting the strengths of private sector firms to commercialize varieties in emerging and competitive markets. NARS then, no longer remain the main technology provider, but continue their engagement in breeding and variety evaluation, while private companies are expected to develop their breeding capacities and generate their own varieties.

PPPs’ plant breeding and seed delivery systems may vary by country, crops and programme, but they generally involve three main core components (Fig 1):

1. Genetic improvement: through the plant breeding component, PPPs provide direct and continuous access to elite breeding materials and/or finished varieties to domestic private companies and public partners, thus relaxing germplasm restrictions imposed by IPR and facilitating germplasm transfer. An important incentive for seed companies to participate in PPPs is their ability to access CGIAR/NARS improved germplasm. SMEs may similarly be able to use elite cultivars from CGIAR/NARS in their own breeding programmes for the development of follow-on innovations, for which they may seek intellectual property protection. Participating domestic firms also benefit from their ability to refresh their variety portfolios, yielding higher margins and economic returns.
2. Variety development and release: PPPs take advantage of the public and private sector infrastructure to extensively evaluate new potential varieties in multilocation testing networks (MTNs). MTNs in PPPs allow partners to identify materials widely and/or locally adapted to production conditions and potential markets before the selection of best-bet hybrids for commercial cultivation. MTNs are expensive and difficult to conduct and small breeding programmes in developing countries are often unable to establish them at a sufficient scale to provide reliable yield information [27]. Therefore, their use provides SMEs essential information about varieties’ performance that is otherwise inaccessible. One key aspect of PPPs is the development of seed production technology and their transfer to partner companies. Some PPPs also register their variety innovations to facilitate hybrids uptake by SMEs.
3. Variety delivery: PPPs foster the integration of dynamic public breeding programmes and private seed delivery systems, making it possible to continuously deploy new varieties extracted from germplasm collections, stimulate varietal replacement and increase crop yields. Often, public partners provide foundation seed to private companies to accelerate commercial seed production, and in some cases, they engage in varieties’ promotion and dissemination. The variety delivery component is fundamental for achieving the impact of plant breeding research in the international agricultural research system.

### The MasAgro maize consortium

MasAgro maize was one of the four components of the Sustainable Modernization of Traditional Agriculture (MasAgro) project, implemented in Mexico by the government of Mexico and CIMMYT between 2011 and 2019. The specific objectives of MasAgro maize were: 1) to develop a strong and diverse national maize seed sector able to produce and deliver improved maize seeds to an additional 1.5 to 3.0 million ha in rainfed zones, 2) increase maize yields in that area from 2.2 to 3.7 t/ha by promoting the use of improved seeds, and 3) increase national rainfed maize production from about 5.0 to 9 million t [30].

The key features of the MasAgro maize consortium included:

1. Development of advanced inbred lines, crosses and final promotional hybrids for direct allocation to private national companies on a non-exclusive and royalty-free basis.
2. Evaluation of advanced germplasm materials and selection of best-bet pre-release hybrids in a multilocation testing network.
3. Variety registration, foundation seed provision, training, and hybrid seed production technology transfer by CIMMYT to seed firms to speed up hybrid uptake.
4. Segmentation and targeting, marketing support in product portfolios design, and easing branding restrictions for facilitating scaled-up marketing.

Table 1 summarizes the main changes implemented by MasAgro maize along the maize seed value chain. The table outlines the key issues faced at each stage, how these issues were addressed and the outputs or anticipated impacts.

**Table 1:** Changes in the maize seed sector in Mexico with the implementation of MasAgro.

Value chain component	Before 2010	Problem/limitation	During MasAgro	Outputs/anticipated impacts
**R&D**	CIMMYT developed intermediate materials for further use by NARS, especially INIFAP, and private companies in the creation of final hybrids.	Public germplasm was available to local seed companies only through NARS.	CIMMYT developed advanced inbred lines and final hybrids for direct use by national seed companies. Double Haploids (DH) and molecular marker technologies used and transferred to seed companies.	Direct and wider access of germplasm by national companies. Enhanced breeding capacities. Potential competition between CIMMYT and NARS in maize breeding. Potential disincentive to private companies for investing in their own R&D pipeline.
**Testing and evaluation**	Testing of public and private materials developed by NARS and seed firms was performed separately. Public NARS cultivars were provided to seed companies for commercial production.	Seed companies did not participate in testing and selection of public hybrids, having limited knowledge of product adaptation and performance.	Public and private hybrids jointly tested by CIMMYT, NARS and private companies in a multilocation testing network and selected for commercial production.	Higher information exchange about product performance. Broader scale evaluation of seed varieties. Products well suited and adapted to local conditions and target markets.
**Registration and certification**	Inbred lines and finished varieties described and registered for plant breeder’s rights by NARS. CIMMYT did not describe and register its seed varieties.	CIMMYT did not protect breeders’ rights in its seed innovations. Lengthy and expensive variety description process for seed firms using public germplasm.	New products developed in MasAgro registered and protected for plant breeders’ rights by CIMMYT. Companies could seek IPRs over MasAgro follow-on innovations.	Faster adoption of new hybrids by seed companies. Registration and certification process for seed firms eased and accelerated.
**Foundation seed production**	Foundation seed was produced by NARS and provided to seed companies for hybrid production and commercialization.	Foundation seed shortages were a major limitation. Firms could not access breeders’ seed.	Foundation seed produced by CIMMYT for provision to seed companies at no cost. NARS continued to produce and sell it to seed firms.	CIMMYT complements NARS’ foundation seed provision. Access to breeders’ seed for enhanced long-term private breeding capacities.
**Seed production**	Seed production technology was available to seed companies from NARS.	Poor and heterogeneous seed quality.	Seed production technology of new hybrids developed and transferred by CIMMYT to seed companies.	Enhanced production capacities of small and medium seed companies. Seed quality improved.
**Marketing and sales**	Seed companies were required to sell public varieties under the name given by the breeding institution.	Heterogeneous quality and risk of marketing the same hybrid. Lack of incentives for brand, product & market development.	Freedom for naming and branding new CIMMYT-germplasm based hybrids.	Higher incentives for brand, product & market development, and product differentiation.

Source: Authors

## Section 3: Data and methods

Despite the large number of PPPs for seed sector development established since the early 1990s, there have been no empirical evaluations that assess their performance. Here, we assess MasAgro maize based on a set of indicators that capture the PPP’s performance in variety development, SMEs varieties’ uptake and variety turnover, and MasAgro maize effect on the maize seed market. Our selection of indicators and methodological approach are discussed below.

### Variety development

Firstly, we classified varieties into five categories based on the entity responsible for their development or the proprietary holder. These categories are 1) MasAgro (hybrids developed by CIMMYT and made available to SMEs through MasAgro), 2) Private MasAgro (hybrids developed by consortium companies using MasAgro lineage), 3) Public (hybrids developed by public NARS), 4) Private national (hybrids developed by national companies using proprietary germplasm), and 5) Multinational (hybrids developed by multinational companies). Then, we used variety releases and variety performance to assess MasAgro maize efficiency in genetic improvement. The number of varieties released and their performance indicate the efficiency of a plant breeding programme, and a seed system’s capacity to deliver new cultivars, and enhance agricultural productivity [31,32].

To analyze the trend in variety releases we used the following data sources:

***The MasAgro seed evaluation network*** [33]: every year, MasAgro maize established a seed evaluation network to evaluate all materials developed by CIMMYT breeders, and assess their performance vis-à-vis public and private materials under development or readily available in the market. Therefore, this dataset contains observations of new hybrids developed by MasAgro maize as well as the yield performance of MasAgro, public, private national, and multinational cultivars evaluated in 2011–2019.

***The MasAgro seed marketing survey*** [34]: this survey was administered from 2013 to 2019 to MasAgro project affiliated SMEs to monitor their seed sales, the uptake of new MasAgro hybrids, and the number of private MasAgro hybrids developed by affiliated firms. The number of companies that responded to the seed marketing survey each year was: 2013 (n = 29), 2014 (n = 24), 2015 (n = 39), 2016 (n = 42), 2017 (n = 42), 2018 (n = 52), 2019 (n = 61).

***The National Seed Varieties Catalogue (CNVV)*** [35]: the CNVV is the official varietal releases database of the Mexican government. It contains the number of varieties developed and released by NARS, private national and multinational companies, and registered for production and commercialization in the National Seed Inspection and Certification Agency (SNICS).

Variety performance was assessed comparing grain yield (GY) of MasAgro, Public, Private national and Multinational hybrids using data from [33]. The original database contained N = 897 observations. These data were previously processed and analyzed by CIMMYT’s Subtropical, Tropical and Highlands maize breeding programmes as described by [36]. However, we used a sub-dataset (n = 341) containing GY of the top two white and two yellow hybrids evaluated in each mega-environment in 2011–2019. We only selected the two best hybrids for each category, colour and mega-environment, given that only the top one or two commercial multinational checks were used in the MasAgro seed trials network each year. The number of private MasAgro hybrids and open-pollinated varieties (OPVs) in the MasAgro multilocation trials was too little to be included in the analysis. OPVs were also excluded to compare only hybrids. Therefore, we compared GY across all years (2011–2019), for each colour and mega-environment using OLS regression.

The linear regression model used was:


$${GY}_{i}=\beta_{0}+\beta_{1}{ORI}_{i}+\beta_{2}{ORI}_{i}*{MEGA}_{i}*{COL}_{i}+\beta_{3}{Year}_{i}+\varepsilon_{i\;}\;$$ (1)

Where the dependent variable (${GY}_{i})$ is the grain yield response (t/ha) of the *ith* hybrid; ${ORI}_{i}$ captures the origin of the *ith* hybrid (MasAgro, private national, multinational, public; being the latter the baseline); ${MEGA}_{i}$ and ${COL}_{i}$ capture the mega-environment (subtropic, tropic, and highlands; being the latter the baseline) and colour (white or yellow; being the latter the baseline) for the *ith* hybrid respectively. ${ORI}_{i}*{MEGA}_{i}*{COL}_{i}$ is an interaction term of the three variables, to capture the combined effects. ${Year}_{i}$ is the evaluation year of the *ith* hybrid; and $\varepsilon_{i}$ captures the random error assumed to be normally distributed.

In addition to the regression estimations, we used the interaction term to compute the marginal effects of the interaction between origin, environment, and colour on the grain yield response, and report the results as a matrix of statistical differences between effects. The analysis was made using Stata 17.

### Varieties’ uptake and variety turnover

We measured varieties uptake and variety turnover among a sample (n = 31) of MasAgro SMEs. Varieties uptake and variety turnover denotes SMEs innovation capacity and ability to integrate and exploit external knowledge and resources (e.g., improved germplasm, new varieties) into their operations. The greater is this capacity, the greater are the expected outcomes in product innovation, e.g., developing and launching new products, incorporating new hybrids into product portfolios, and expanding seed sales [25]. The uptake of new varieties from breeding organizations by SMEs translates into higher number of commercial varieties available for farmers. In turn, a rapid rate of varietal turnover defines the rate at which companies replace old cultivars with new ones [27], and it is associated with faster variety replacement at farmers’ fields [37] and a competitive seed sector [27].

Drawing upon research on SMEs performance from the management literature, we assessed varieties uptake and variety turnover among our sample of SMEs. We did so using the following indicators: 1) New-to-firm and new-to-market varieties introduced by SMEs into their product portfolios [38,39]; 2) Sales of new MasAgro hybrids relative to other categories and the total firm’s sales; 3) The number of follow-on products developed using MasAgro lineage [39,40]; and 4) Product portfolio turnover for the period 2011–2019 [38]. The data were obtained from four different sources. The first was [34]. The second is a cross-sectional survey [41] applied by CIMMYT to thirty-nine MasAgro seed companies in 2015, to collect information of all seed varieties sold by affiliated businesses. This survey contains, inter alia, variety-wise information of 2014 seed sales, seed origin, product type, seed colour and first year of product introduction to the market. These datasets were combined into a database of thirty-eight companies having at least six to seven years of variety-wise sales information. To these thirty-eight firms, a new survey (the product portfolio survey) was distributed in 2020 to cover existing information gaps, specially the 2011–2012 seed sales, company size (seed sales and number of employees) and each product’s first year of introduction to the market. Out of all firms contacted, seven did not respond, leaving us a sample of 31 companies with information on seed sales at the variety level [42]. All surveys were handled via e-mail by the MasAgro maize direction in the CIMMYT Global Maize Program. By answering the survey, seed companies agreed to participate in the study in line with a collaboration agreement with MasAgro maize. To protect the confidentiality of our sample companies, all personal and commercial information on private companies was made confidential.

Table 2 shows our final sample composition and relevant descriptive statistics. We classified sampled companies in three groups based on 1) maize seed sales, as medium (≥ 1,000−5,000 t of seed sales), small (≥ 200−1,000 t) and micro enterprises (<200 t); 2) the number of employees, following the guidelines of the North American Industrial Classification of Economic Activities (SCIAN) [43], and 3) breeding research capacities, i.e., whether the firm had an established breeding research programme, reflected on its product’s portfolio by having own proprietary hybrids.

**Table 2:** Overview of MasAgro sampled seed companies (n = 31).

Company size	Firms	Mean years in operation	Mean number of employees	Mean seed sales (2011–2019)		Maize varieties by germplasm type (%)		
				t	%	Private	Private with public^a^	Public^b^
Medium	5	26.2	82.6	5,954	54.2	41.1	21.2	37.7
Small	8	14.9	22.9	3,183	29.0	6.6	11.6	81.8
Micro	18	14.6	8.2	1,840	16.8	14.6	10.1	75.3
Total/average	31	16.5	24.5	10,977	100.0	20.8	14.3	64.9

Source: [34,41,42].

^a^Own proprietary hybrids containing one or more inbred lines or improved populations from CIMMYT or NARS.

^b^Includes hybrids developed in CIMMYT for MasAgro.

Micro enterprises comprised more than half of the seed firms sampled. Medium companies had almost twice as long as small and micro seed businesses in the market. They employed four times more staff than small firms and ten times more than micro seed enterprises. Medium seed companies accounted for the largest share of sales (54%), whereas small businesses participated with 29% and micro with 17%. Based on the proportion of varieties by germplasm type, it is evident that medium-sized companies had more proprietary hybrids, unlike small and micro seed firms that depended largely on public materials to sustain their portfolios.

We estimate that MasAgro affiliated companies account for approximately 85–90% of the total market share captured by national companies. Our sample represents about 62% of maize seed sales of all MasAgro affiliated businesses, and nearly 60% of sales of national companies in the maize seed industry.

### MasAgro maize effect on the maize seed market

The information on seed sales of our sample of MasAgro companies was complemented with seed production statistics provided by the SNICS [44]. These data contained variety-wise information on national non-MasAgro and multinational firms for the 2011–2015 and 2019 sales years. With these data, we compiled a final database [45] showing detailed seed production and sales figures of the entire maize seed industry for our study period. These data have two limitations: 1) not all national companies register their seed production with the SNICS, a drawback that was compensated by the inclusion of variety-wise information on domestic companies available from MasAgro, and 2) variety-wise information on 2016, 2017 and part of 2018 sales year seasons for non-MasAgro companies was unavailable, and therefore, the market shares for these years contains some estimations. Despite these limitations, we consider this data as a unique dataset, given the difficulties of collecting variety-wise production and sales statistics from the private sector.

Finally, using data from [45], we estimated the market share of our different seed categories, and the degree of market concentration in the maize seed industry. We calculated three concentration indexes: the Hirchman-Herfindahl Index (HHI), Gini Coefficient (Gr), and Entropy Index (EI). The HHI index is the sum of squared market shares of all firms in the market. Squaring the market shares places greater weight on companies with large market shares, and neglects concentration from multiple small firms which together may account for a large proportion of the market (i.e., market share inequality). The Gr and the EI complement the HHI by accounting for inequality of shares across all companies and the effect of small firms on the market [46–48]. The values for the three indexes range from 0 to 1. Larger HHI and Gr values indicate greater concentration, and lower values approaching to 0 indicate quasi-perfect competition. Markets with HHI > 0.25 and Gr > 0.5 are considered highly concentrated. The EI is inversely related to the HHI and GR indexes as low values indicate high industry concentration [31,49]. The procedure to calculate each of these indexes can be found in [46–49].

## Section 4: Study context

### Maize production in Mexico

In Mexico, out of the total 7.4 million ha planted to maize, about 5.9 million ha (79%) is rainfed. The country produces 27.4 million t of maize annually [50] and is the world’s eighth-largest maize producer [51]. However, it depends substantially on imports (about 37%) to meet its national consumption needs [52]. In 2020, Mexico became the first worldwide maize importer [53]. This deficit is partly explained by the predominant low crop yields, resulting from a predominantly rainfed maize production system, and the poor adoption rates of improved seeds (40%) [50,54].

**Fig 2:**
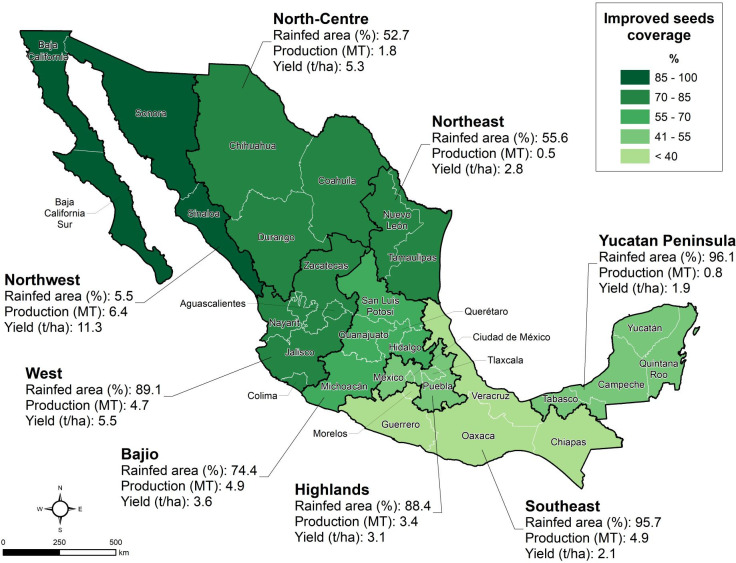
Improved maize seeds coverage, maize rainfed area, maize production and yields by commercial maize seed region in Mexico. Source: Authors, generated in ArcGIS using state boundaries from [55] and data from [50,54].

Maize yields range from an average of 2.4 t/ha in rainfed to 8.7 t/ha in irrigated areas [50]. However, grain yields vary significantly among regions (Fig 2). Most farmers in the Northwest obtain yields above 10 t/ha because of the high rates of improved seeds, fertilizers use, planting densities and irrigation. In the West and North-Centre, farmers harvest above 5.0 t/ha as improved seeds coverage is estimated at 82% and 72% respectively. In these regions, seed markets have been well developed in response to the commercial irrigated and good potential rainfed areas which represent segments of high value for seed companies, especially multinational. In contrast, in the states of the Southeast, Yucatan Peninsula and the Highlands, yields are below 3.0 t/ha as maize is mostly grown in traditional rainfed systems associated with own consumption, low-technology small-scale farming, with limited use of improved varieties and fertilizers. In these regions, there are about 2.56 million ha where maize production and yields can be doubled with the use of improved seeds delivered by PPPs.

### The maize seed industry

**Fig 3:**
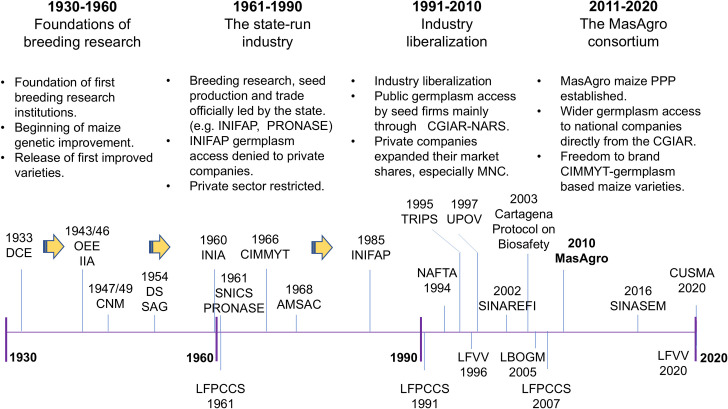
Timeline of the evolution of the Mexican maize seed industry, 1930–2020. Department of Experimental Stations (DCE), Office of Special Studies (OEE), Institute of Agricultural Research (IIA), National Maize Commission (CNM), Seed Department of the General Office of Agriculture (DS-SAG), National Institute of Agricultural Research (INIA), National Seed Production Company (PRONASE), National System for Seeds Inspection and Certification (SNICS), International Maize and Wheat Improvement Centre (CIMMYT), Mexican Seed Producers Association A.C. (AMSAC), National Institute of Forestry, Agriculture and Livestock Research (INIFAP), North American Free Trade Agreement (NAFTA), Agreement on Trade-Related Aspects of Intellectual Property Rights (TRIPs), International Union for the Protection of New Varieties of Plants (UPOV), National System of Plant Genetic Resources for Food and Agriculture (SINAREFI), Cartagena Protocol on Biosafety to the Convention on Biological Diversity (Cartagena Protocol on Biosafety), Sustainable Modernization of Traditional Agriculture (MasAgro), National Seed System (SINASEM), Canada-United States-Mexico Agreement (CUSMA), Law of Production, Certification and Commercialization of Seeds (LFPCCS 1961, 1991 and 2007), Federal Law of Plant Varieties (LFVV 1996 and 2020), Genetically Modified Crops and Biosafety Law (LBOGM). Source: Authors.

Between 1930 and 1960, the government of Mexico created several state breeding research institutions which have evolved into the current national agricultural research system (Fig 3). This process started with the creation of the Department of Experimental Stations (DCE) and the establishment of the first formal breeding research programmes in 1933. Breeding intensified with the establishment of the Office of Special Studies (OEE) – currently CIMMYT – in 1943, and the evolution of the Department of Experimental Stations (DCE) into the Institute of Agricultural Research (IIA) – currently the National Institute of Forestry, Agriculture and Livestock Research (INIFAP) – in 1946. As a result, the first improved varieties were released in 1947, and self-sufficiency in corn, beans and wheat was achieved in 1959 [56].

The exceptional success of breeding research during the early years of the maize seed industry led the government to enact the first seed law in 1961. This law restricted private sector participation in the maize seed industry. Seed companies could not access public germplasm developed by NARS and could not carry out breeding research without permission from the state. Seed imports and exports were controlled. There were also restrictions on seed marketing, and seed prices were set up for the private sector while public seed from the parastatal seed company (PRONASE) was subsidized [57,58]. Despite many restrictions imposed on the private sector, private companies – mainly multinational – have been actively engaged in the maize seed industry since the 1960s [59] and have consistently captured a substantial share of the maize seed market since the 1970s. Between 1970 and 1989, private companies maintained on average a 53% share of the maize seed market. After liberalization of the seed industry in 1991, the private sector increased its share from 58% to 87% in 1996. The public sector accounted for 13% of the maize seed market, mainly through the government-backed company PRONASE [58].

**Fig 4:**
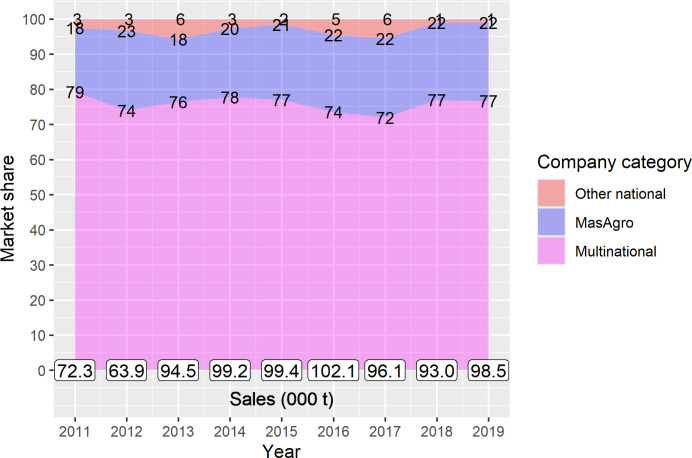
Market shares of multinational and national seed companies in the Mexican maize seed industry, 2011–2019. Source: [45]. Seed sales of multinational companies were adjusted using official seed imports and exports from [60].

With liberalization of the maize seed industry the maize seed market in Mexico has also over time become highly concentrated. Over the period 2011–2019, private companies marketed on average 90,963 t annually (4.55 million bags of sixty-thousand seeds) of improved maize seed (Fig 4). In 2019, domestic companies had a share of 23%, while multinationals captured over 75% of the maize seed market. One single firm (Bayer) concentrated 60% of total seed sales [45].

MasAgro maize can be seen as a response to the challenges faced in the maize seed industry and experienced in the CGIAR-NARS model of variety development and dissemination, reflected in the limited uptake of improved varieties in only 40% of the maize area in Mexico, notwithstanding the huge public investments in plant breeding made over several decades.

## Section 5: Results

### Variety development

**Fig 5:**
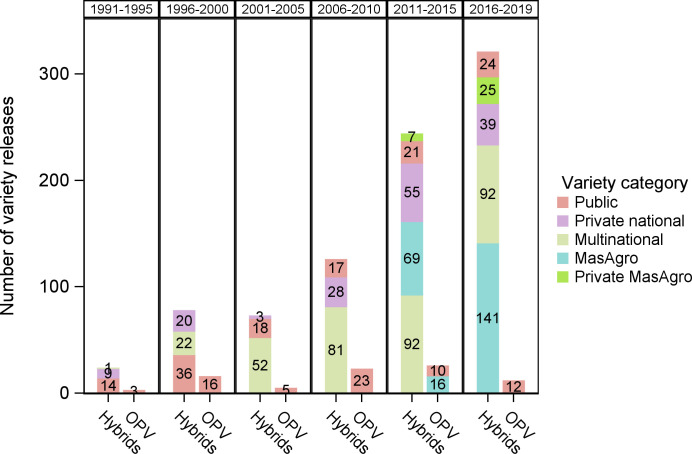
Maize seed variety releases in Mexico, 1991–2019. Source: [33–35].

**Fig 6:**
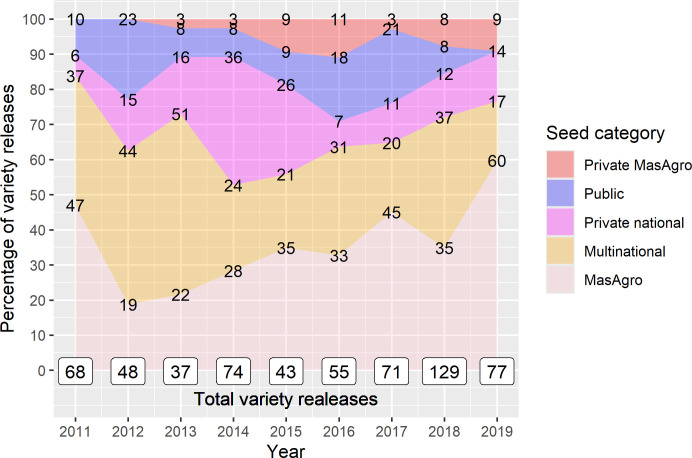
Maize seed variety releases by CIMMYT for MasAgro, public NARS, national and multinational seed companies, 2011–2019. Source: [33–35].

MasAgro maize developed a total of 226 maize hybrids and OPVs during 2011–2019 for allocation to private companies. Varieties developed were predominantly three-way white hybrids for the tropics (39%), highlands (35%) and subtropics (26%). OPVs accounted for a small proportion of varieties developed (Table 3). MasAgro maize similarly developed a greater number of seed varieties than INIFAP and other NARS together in a significantly shorter period of time (Fig 5). During the period 2011–2019, MasAgro and multinational companies were the main sources of maize seed varieties (Fig 6).

**Table 3:** MasAgro hybrid maize release records, 2011–2019.

Mega-environment	Hybrids developed	Cross-type					
		Single		Triple		OPVs	
		White	Yellow	White	Yellow	White	Yellow
Subtropical	60	4	8	29	19	–	–
Tropical	87	15	14	30	25	3	–
Highlands	79	9	6	27	24	12	1
*Subtotal*		*28*	*28*	*86*	*68*	*15*	*1*
Total	226		56		154		16

Source: [33].

Table 4 presents the results of the linear regression model estimating GY across all years (2011–2019), seed categories, colour and mega-environment. The estimated coefficients for both, MasAgro and multinational hybrids, are positive and significant, indicating they had higher yields than public varieties. The estimated coefficients for Subtropics and White are also positive and significant, showing a GY advantage in this mega-environment and white hybrids. The interaction effect between origin and mega-environment, origin and colour, and origin, colour, and mega-environment shows a significantly lower GY of private national hybrids compared to public varieties in the Tropics, a lower GY of multinational white hybrids compared to their counterpart yellow hybrids, and a higher GY of multinational white hybrids over public varieties in the Tropics. In general, all breeding programs showed a significant increase in GY since 2014.

**Table 4:** GY regression coefficients, standard errors, and significance levels of MasAgro, multinational, private national and public hybrids evaluated in the MasAgro seed evaluation networks in Mexico, 2011–2019.

	Coefficient	S.E.
Origin (baseline: Public)		
MasAgro	0.809***	0.288
Multinational	1.141**	0.493
Private national	0.639	0.495
Mega-environment (baseline: Highlands)		
Subtropics	1.223***	0.437
Tropics	0.270	0.455
Origin and mega-environment		
MasAgro#Subtropics	0.142	0.492
MasAgro#Tropics	−0.482	0.487
Multinational#Subtropic	0.638	0.658
Multinational#Tropic	−0.929	0.641
Private national#Subtropic	0.452	0.621
Private national#Tropic	−1.181*	0.642
Colour (baseline: Yellow)		
White	1.058***	0.342
Origin and colour		
MasAgro#White	−0.667	0.419
Multinational#White	−1.123*	0.617
Private national#White	−0.480	0.586
Mega-environment and colour		
Subtropic#White	−0.266	0.519
Tropic#White	−0.705	0.538
Origin, colour, and mega-environment		
MasAgro#Subtropic#White	0.067	0.637
MasAgro#Tropic#White	0.997	0.621
Multinational#Subtropic#White	0.256	0.789
Multinational#Tropic#White	1.345*	0.797
Private national#Subtropic#White	0.193	0.736
Private national#Tropic#White	1.037	0.768
Year		
2012	−0.316	0.213
2013	0.143	0.218
2014	1.178***	0.187
2015	0.642***	0.198
2016	1.456***	0.212
2017	1.187***	0.213
2018	1.739***	0.262
2019	0.960***	0.222
Constant	5.045***	0.288
R-squared	0.704	
F-test	45.91***	
Sample	341	

Robust standard error *** p < 0.01, ** p < 0.05, * p < 0.1. Source: [33].

**Fig 7:**
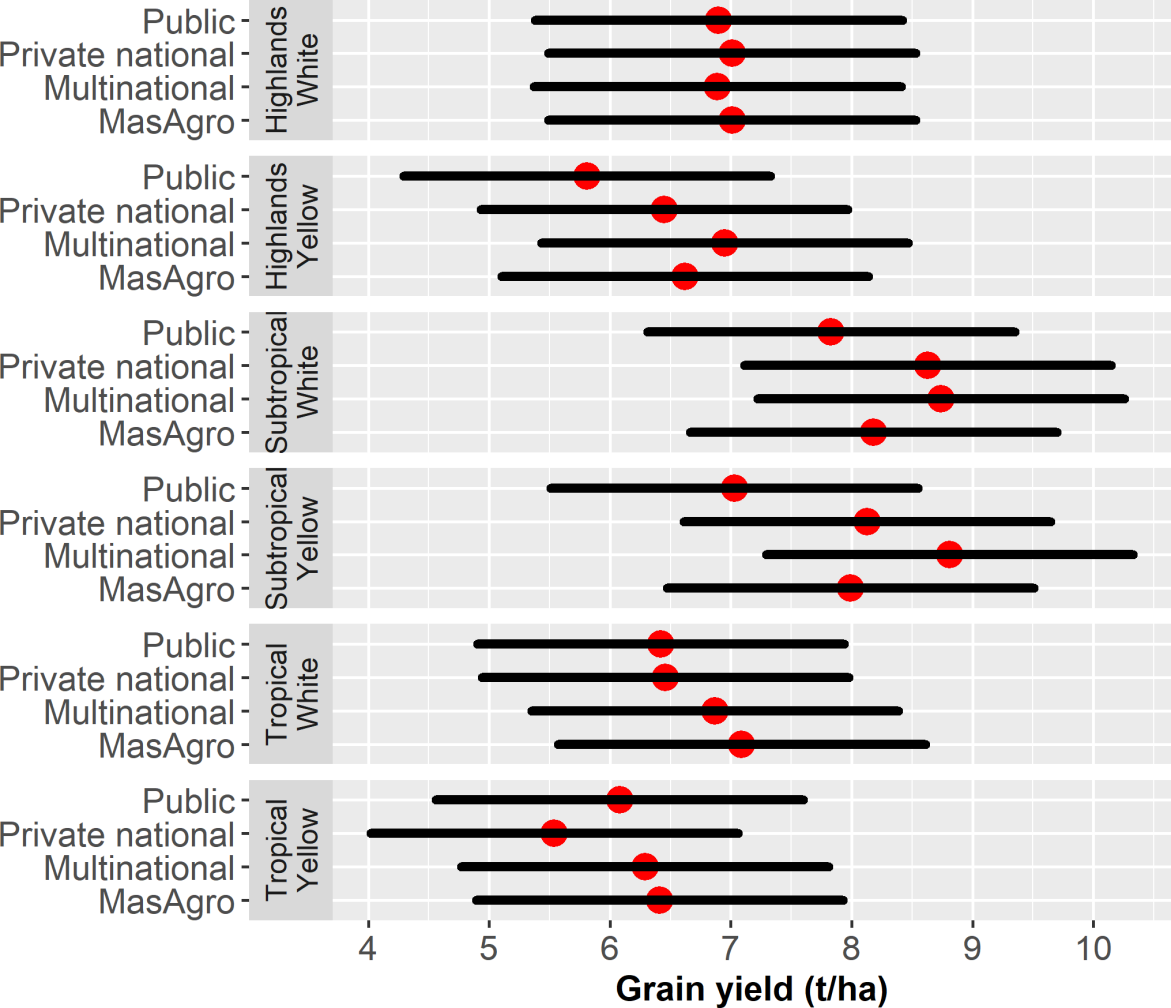
Average grain yield (t/ha) of MasAgro, multinational, private national and public hybrids evaluated in the Highlands, Subtropical and Tropical MasAgro seed evaluation networks in 2011–2019. Source: [33].

Fig 7 illustrates the GY (t/ha) comparison of white and yellow MasAgro, multinational, private national and public hybrids tested in the MasAgro seed evaluation network from 2011 to 2019. Mean GY results, GY differences, standard errors, and p-values across categories were obtained by estimating the marginal effects of the mean and are shown in S1 Table. Overall, subtropical hybrids showed the highest GY (average 8.34 t/ha white and 7.99 t/ha yellow) among all mega-environments. In this mega-environment, multinational white and yellow hybrids maintained a yield advantage over MasAgro of about 0.5 t/ha and 0.8 t/ha (p < 0.05) respectively. Private national materials similarly showed higher yields than MasAgro although differences between yellow hybrids were too small and not significant.

In the Highlands, private national white hybrids had the highest yield (7.03 t/ha) followed by MasAgro, multinational and public hybrids, but no significant difference was observed. For yellow maize, multinational hybrids had the highest yield (6.95 t/ha) but there was no significant difference with MasAgro and private national materials. Finally, white and yellow MasAgro hybrids in the Tropics outperformed all other categories with an average GY of 7.09 t/ha and 6.41 t/ha respectively. For both white and yellow hybrids, there was no significant difference between tropical MasAgro and multinational hybrids.

The large number of hybrids developed by MasAgro represented an opportunity for domestic seed firms to modernize their seed offering and expand their market shares. In the next section we examine the uptake and commercialization of MasAgro hybrids by our sample consortium companies (n = 31), and the impact of the introduction of MasAgro hybrids on the product portfolios and sales composition of these companies.

### Varieties’ uptake and variety turnover

#### Product portfolios composition.

**Fig 8:**
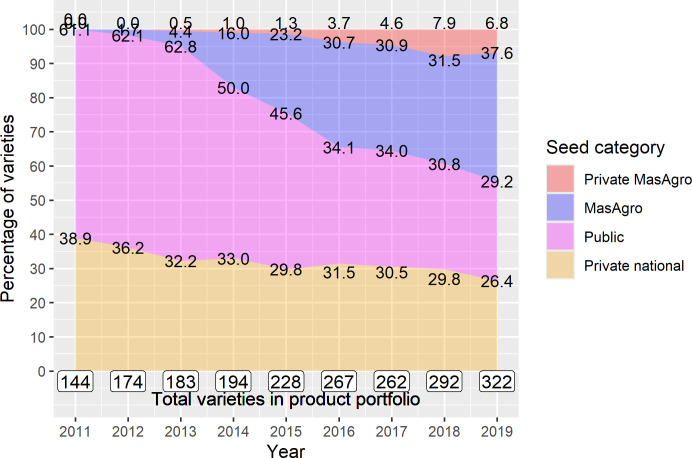
Product portfolio composition of maize varieties, 2011–2019 (n = 31). Source: [34,41,42].

**Fig 9:**
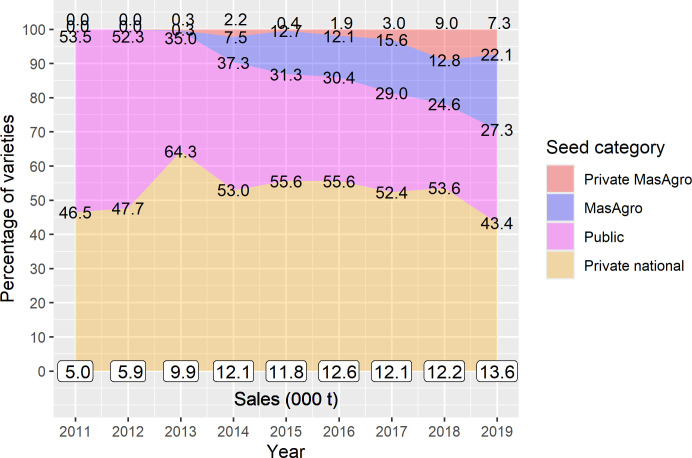
Composition of maize seed sales by seed category, 2011–2019 (n = 31). Source: [34,41,42].

In 2019, sampled companies sold more than double the number of seed varieties they sold in 2011. Out of the total varieties, 121 (37%) were MasAgro and 22 (6.8%) were private MasAgro, i.e., hybrids developed by affiliated companies using MasAgro lineage (Fig 8). Overall, seed sales of sampled companies also increased almost threefold (Fig 9): by 2019, private national hybrids represented 43% of total sales, MasAgro and private MasAgro hybrids represented 22% and 7.3% respectively, and the share of public varieties fell from 53% in 2011 to 27% in 2019. The same MasAgro hybrid may have been sold under different brand names/nomenclatures by different companies. Similarly, the same public variety could have been sold by two companies under the same name. That said, all the MasAgro varieties derived from 41 hybrids developed by CIMMYT for MasAgro; and Public varieties resulted from 44 varieties developed by INIFAP and 9 by other NARS.

S1–S6 Figs show the number of maize varieties and the percentage of seed sales by product type (hybrid or OPV), mega-environment and company size. The number of hybrids in companies’ portfolios almost tripled while OPVs remained stable (S1 Fig). Seed sales of OPVs decreased from 28% to 8%, in contrast to hybrids whose share expanded from 72% to 92% (S2 Fig). The number of products by mega-environment more than doubled in all categories (S3 Fig), but sales of subtropical hybrids still accounted for most of the total sales by 2019, suggesting companies did not move from their traditional subtropical markets towards markets for tropical and highland varieties (S4 Fig).

Finally, medium-sized firms relied mostly on their own hybrids, with an average of 84% of seed sales over 2011–2019 coming from proprietary sources. Yet by 2019, they introduced up to 17 MasAgro hybrids, which represented 12% of their seed sales (S5a and S6a Figs). Small and micro seed businesses, in contrast, remained heavily dependent on public breeding research. Together, public and MasAgro materials represented around 76% of total products and sales for small companies over the study period. They introduced up to 45 MasAgro hybrids, which by 2019 accounted for 28% of their seed sales, while their share of public varieties dropped from 84% to 40% (S5b and S6b Figs). As for micro seed businesses, sales of public varieties declined from 73% in 2011 to 50% in 2019. These companies introduced 59 new hybrids from MasAgro (almost half out of the total 121), which by 2019 accounted for 35% of their total sales (S5c and S6c Figs).

#### Product portfolios turnover.

**Fig 10:**
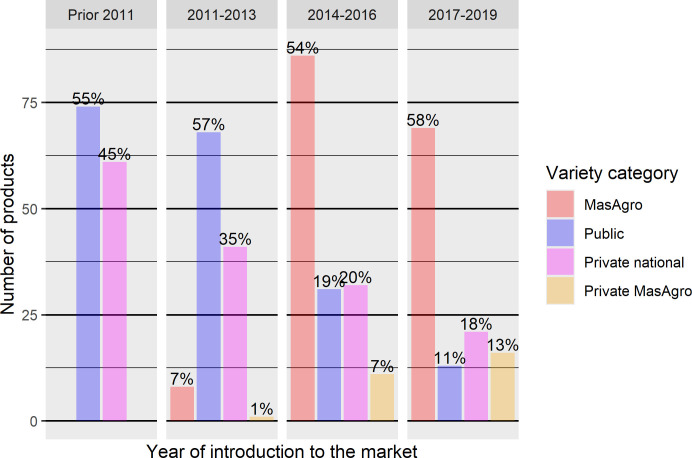
Product portfolio composition of maize varieties by germplasm source and year of introduction to the market, 2011–2019 (n = 31). Source: [34,41,42].

During 2011–2019, a total of 532 seed varieties were sold. MasAgro and private national hybrids represented on average 56% and 19% respectively of the products introduced in 2014–2019. On the other hand, the products launched between 2011–2013 and earlier were mainly public and private national varieties (Fig 10).

The number of seed varieties in product portfolios and the percentage of sales by the age of varieties for 2011–2019 are shown in Table 5. The number of products in all age groups increased as a result of product portfolio expansion. On average, varieties older than 9 years represented about 30% of the total varieties. In terms of seed sales, the share of varieties aged over 9 years increased from 20% in 2011 to 32% in 2019, varieties aged 4–9 years decreased from -70% to 48%, and varieties launched in the last three years increased from 10% to 20%.

**Table 5:** Product portfolio composition of maize varieties and composition of maize seed sales by varieties’ year of introduction to the market, 2011–2019 (n = 31).

Variety age	2011	2012	2013	2014	2015	2016	2017	2018	2019
Products in portfolio								
0-3 years	38	72	91	43	85	133	34	60	103
4-6 years	54	54	44	59	61	56	102	114	104
7-9 years	37	34	30	47	35	33	52	50	53
10-12 years	6	6	6	28	28	25	33	30	29
13-15 years	2	2	4	7	5	5	23	21	20
>15 years	7	6	8	10	14	15	18	17	13
***Total***	***144***	***174***	***183***	***194***	***228***	***267***	***262***	***292***	***322***
	Percentage of sales								
0-3 years	10.18	18.76	30.67	12.84	21.81	21.49	3.00	8.55	20.39
4-6 years	41.73	43.97	24.76	21.65	33.24	37.84	24.17	27.54	25.00
7-9 years	27.97	18.02	12.30	29.55	13.35	15.73	35.06	26.73	22.67
10-12 years	4.79	1.19	2.81	11.43	11.19	9.37	13.71	15.63	13.33
13-15 years	6.22	5.20	6.63	1.60	1.74	1.05	9.68	8.86	10.12
>15 years	9.12	12.85	22.82	22.93	18.68	14.53	14.38	12.69	8.48
***Total***	***100***	***100***	***100***	***100***	***100***	***100***	***100***	***100***	***100***

Source: [34,41,42].

### MasAgro maize effect on the maize seed market

**Fig 11:**
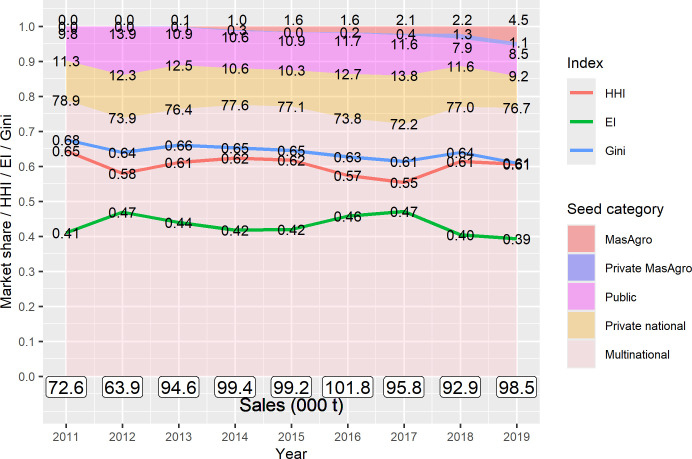
Market shares of MasAgro, private MasAgro, public, private national and multinational hybrids, and concentration indexes in the Mexican maize seed market, 2011–2019. Source: [45].

Fig 11 shows market shares of MasAgro, private MasAgro, public, private national and multinational hybrids, and concentration indexes obtained from seed production and sales figures of the entire maize seed industry in Mexico for the period 2011–2019. As of 2019, 48 affiliated companies commercialized 47 MasAgro and 32 private MasAgro hybrids. By 2019 these hybrids accounted for 5.6% of total maize seed sales. All other seed categories experienced a slight decline in market shares between 2011 and 2019: public varieties dropped from 9.8% to 8.5%, private national from 11.3% to 9.2% and multinational from 78.9% to 76.7%. Nevertheless, multinational hybrids consistently maintained their leadership in the maize seed market, accounting for roughly 77% of total maize seed sales. Concentration in the maize seed industry slightly declined from 2011 to 2019 as shown by market shares and the HHI, Gini and EI concentration indexes, but the maize market remained highly concentrated.

## Section 6: Discussion

Our analysis shows that the direct transfer of elite breeding materials and finished maize hybrids directly by CIMMYT to domestic companies was very successful in generating a large number of agronomically competitive commercial hybrids between 2011 and 2019. Considering the figures of maize variety releases of the SNICS [35], the number of hybrids brought to the market through MasAgro substantially outstripped the total of hybrids generated by multinational companies during the last ten years, and was nearly double the number of varieties released by INIFAP in the last 30 years [see 61]. Across several years of multilocational variety trials, MasAgro hybrids out-yielded public, private national and private multinational white and yellow seed varieties in the Tropics and the Highlands. In the Subtropics, multinational and national companies were established more than fifty years ago while CIMMYT subtropical breeding programme was discontinued and re-started operations in 2010, which helps to explain the advantage of private varieties over MasAgro. Seed companies responded positively by successfully incorporating new hybrids into their seed offer and the market and replacing OPVs. The project was of special relevance for small (≥ 200−1,000 t sales) and micro seed companies (<200 t), which in 2019 introduced 50% and 37% respectively of the total hybrids developed by MasAgro maize.

The commercialization of a large number of hybrids within a relatively short time shows that MasAgro maize was successful in leveraging CIMMYT’s strength in plant breeding with the marketing agility of seed firms in the private sector. Specifically, the consortium provided seed companies with 1) a wider, direct, and continuous access to breeders’ seed and advanced germplasm generated by CIMMYT breeding programmes adapted to target specific environments and local markets; 2) prompt and opportune access to quality foundation seed; 3) training and transfer of breeding and seed production technology; and 4) support in product portfolios design as well as the ability to brand public CIMMYT germplasm-based hybrids. These key elements were supported by a targeting and segmentation strategy to orient sales towards areas with the lowest yield and greatest immediate potential impact to encourage rapid dissemination of new hybrids. As observed from year-on-year increases in the number of private hybrids, the provision of inbred lines at no cost or royalty-free to seed companies did not disincentivized R&D investment amongst domestic seed companies.

Despite the successful adoption of new MasAgro hybrids by seed companies, by 2019 a fifth of varieties in the consortium firms’ product portfolios was over 9 years old, representing 32% of their seed sales. The retention of a substantial share of older varieties may reflect the challenges that seed companies face in scaling up the production of seeds of new cultivars, and in popularizing new seeds among farmers when yield and agronomic advantages over their current varieties are only slight [62]. Where hybrid seeds are concerned, firms may also face a steep learning curve in switching to the production of complex new hybrids. Assured and relatively stable markets for existing popular hybrids among farmers may also reduce the incentives for seed firms to bring about rapid variety turnover [27] despite the yield and agronomic advantages of new hybrids over old ones. However, the experience of MasAgro maize shows that SMEs have the willingness and capability for rapid introduction of new hybrids, when access to elite material and foundation seed is guaranteed along with technical training for seed production and enabling seed policies, even in concentrated markets with fierce competition from multinational companies.

MasAgro maize appears to have brought about a significant shift away from public varieties among participating firms towards MasAgro hybrids. Although INIFAP and other public research institutions continued to develop improved seeds at approximately the same pace as during the previous twenty years [35], variety sourcing from NARS dropped by more than 40% (Fig 10). Besides, none of the public maize varieties released by NARS in the previous ten years appears to have been taken up by the consortium companies. Also, the number of private varieties developed from MasAgro lineage accounted for a small share of seed sales of consortium firms, reflecting the limited success of the PPP in stimulating follow-on variety innovations.

This is a matter of concern for the development of the domestic seed sector in Mexico for two reasons. Firstly, because micro and small seed companies do not have the capacity to develop their own proprietary hybrids, neither to produce foundation seed for the production of certified seed of new varieties. This means that SMEs will continue to depend on external sources of germplasm and hybrids, and may not be able to sustain their growth if interventions such as MasAgro are no longer available. Experience in Africa shows that the private sector can produce and supply high-quality foundation seed to SMEs. For example, the AATF founded QualiBasic (QBS) Seed Company to produce and supply foundation seed for SMEs in Kenya, Zambia and South Africa since 2017 [63,64]. In Nigeria, Premier Seeds and Value Seeds produce basic seed for maize hybrids. For other crops such as beans, soya beans, groundnut, cowpea and millet, MultiSeed Company (MUSECO) and Doun Ka Fa produce foundation seed in Malawi, and Mali respectively [65]. This shows the unexploited potential within the private sector in Mexico to enhance foundation seed supply, and reduce SMEs dependency on public external sources of germplasm in the absence of interventions such as MasAgro. Secondly, because except for variety testing, the consortium largely worked independently of public institutions that historically led breeding research in the maize seed industry. While this allowed CIMMYT to make a greater impact in a relatively short period of time, this represents a risk for the sustainability of the national agricultural research system. Long-term sustainability of developing countries’ national breeding research systems can only be achieved if NARS are an active partner in seed sector PPPs in all stages of germplasm improvement, evaluation, and delivery.

MasAgro maize and the changes introduced to the maize seed industry made important contributions to reinvigorating the growth of domestic seed companies. Over a longer period of time, our results corroborate the MasAgro consortium’s role in reversing decades of stagnation of the national maize seed sector as suggested by [66] and [25]. Nevertheless, multinational companies maintained their leadership, and seed sales in the maize seed market remained highly concentrated. For over decades [59], multinationals in Mexico have built a competitive advantage over SMEs, largely explained by their strong financial, commercial and marketing capacities. In contrast, SMEs face several challenges to scaling up the uptake of new hybrids, including the lack of or poor infrastructure for seed production, processing and conditioning; poor infrastructure in remote markets that makes seed distribution difficult; and inappropriate or non-existent seed promotion and marketing strategies [67–69].

Another contributing factor to the low market share of MasAgro hybrids may have been the lack of awareness of new varieties and the low diffusion of hybrids in emerging rainfed markets. Affiliated companies did not expand their seed sales in rainfed regions, and very likely directed most of their efforts to irrigated markets where production increased from 7.6 to 14.2 million t and yield from 4.4 to 8.8 t/ha compared to an increase from 9.9 to 12.9 million t and 1.6 to 2.5 t/ha in rainfed zones [50]. This may reflect the limited capacities (or reluctance) of SMEs to move away from mature markets in irrigated areas, and invest in emerging and potential rainfed maize seed markets. Also, domestic SMEs made little use of the MasAgro hubs, the MasAgro programme’s infrastructure designed to disseminate new hybrids and other sustainable farming practices. The basic principle of the hubs [70] was that all actors in the maize seed value chain could interact and create strategic networks and alliances facilitating long-lasting technology adoption processes. The hubs included a research platform, where new technologies were tested and validated by scientists; farmer modules, where farmers and technicians demonstrated the benefits of the new technology on on-farm comparative field trials; and extension areas, where adopters extended the use of innovations beyond farmer modules into their own-managed plots.

The hubs were an opportunity to link MasAgro maize and SMEs research findings from on-station trials to farmers’ fields through, for example, the establishment of demonstration plots, field days and trade shows to showcase the benefits of new hybrids, enable farmers to observe and learn about their performance and attributes, and facilitate the interaction between seed producer SMEs and potential customers (input suppliers and farmers) [71]. Along with product demonstrations, the distribution of free seed samples could have facilitated farmers to gain first-hand, direct experience with new hybrids and accelerate dissemination [72,73]. Furthermore, the hubs could have been used as a platform to leverage farmers, extension agents and input suppliers’ engagement in education and training, demonstrations, and marketing campaigns to create awareness and seed demand. However, domestic SMEs did not take advantage of this infrastructure for the implementation of seed promotion and market penetration strategies. The lack of integration between breeding and seed delivery systems, especially with downstream actors such as extension agents, agro-dealers and farmers may have limited the uptake of MasAgro hybrids.

Finally, one of the MasAgro maize objectives was to contribute to the development of a strong and diverse national maize seed sector. It was expected that the provision of direct access to elite breeding material, finished varieties and training in modern breeding technologies, would enable seed companies to develop their own breeding capacities. Affiliated companies developed thirty-two hybrids using MasAgro lineage, suggesting some degree of spillover from public R&D to the private sector, but overall and for our study period, the follow-on plant breeding capacity of sampled firms appears to have been limited.

The experience of MasAgro maize highlights the challenges that PPPs face in accelerating variety development and delivery in the context of similar developing country seed systems. These constraints are, primarily:

a) The limited plant breeding capacity of SMEs and their restricted potential for developing follow-on innovation using elite germplasm accessed from CGIAR centres or NARS. This may perpetuate SMEs dependency on external sources of germplasm for renewing their product portfolios.
b) The inability of SMEs, even if they are part of a PPP, to significantly increase their market shares. Even though PPPs can deliver competitive new varieties, and SMEs bring these cultivars to market, the dominant players may retain their market shares in highly concentrated markets.
c) The lack of market-led interventions in public breeding research, notwithstanding the recognition of marketing as an essential component for seed systems development [58,74]. The key challenge for PPPs is to scale up new hybrids in head-on competition with global market leaders in concentrated maize seed markets. This underlines the need for PPPs to incorporate a commercial and market-oriented perspective that integrates dynamic public breeding programmes and efficient private seed delivery systems.

## Section 7: Conclusion

The experience of MasAgro maize shows that PPPs working with the private domestic seed sector in developing country contexts can be an effective instrument for accelerating dissemination of crop variety innovations developed by international and national public sector research organizations. This is feasible if SMEs are supported on a sustained basis with 1) continuous access to elite germplasm adapted to target specific environments and local markets, 2) extensive evaluation of new cultivars in multilocation testing networks and information exchange about varieties’ performance, 3) a sustained flow of new varieties moving from the breeding component to the seed delivery system through variety registration; 4) prompt and opportune access to quality foundation seed, 5) training and transfer of breeding and seed production technology, and 5) marketing support in product portfolios design, segmentation and targeting. PPPs in plat breeding should include these key elements. However, while PPPs working with private SMEs have strong capacities for developing and commercializing competitive crop varieties, they face significant challenges in marketing these innovations in highly concentrated markets. The development of competitive, high-yielding hybrids alone is insufficient to disrupt concentrated markets, capture market shares from global market leaders, and promote sufficient competition in the seed industry. If PPPs are to succeed in their objective of advancing the development of formal maize seed systems, and bringing affordable quality seed on a large scale to low-income farmers in developing countries, there is an urgent need to incorporate a commercial and market-oriented perspective along all steps of the plant breeding and dissemination process (Fig 1). In addition to these specific design features, governments and policymakers must provide an enabling institutional, marketing and regulatory policy environment. This includes a review and reform of key seed policies to strengthen NARS’ breeding and foundation seed production capacities; ensure NARS collaboration in all stages and components of seed sector PPPs; allow and encourage the production of foundation seed by domestic private companies for its supply to smaller SMEs and other seed producers; accelerate variety release, registration and certification processes; and remove restrictions on branding publicly bred varieties. PPPs may be part of a broader, long-term (e.g., 10–30 years) seed sector development strategy, which ensures consistent funding and capacity creation to consolidate SMEs, promote competitive seed markets, and increase crop productivity gains.

## Supporting information

S1 Appendix Examples of public-private partnerships (PPPs) in plant breeding. (DOCX)

S1 Table GY mean results, GY differences, significance levels and standard errors of MasAgro, multinational, private national and public hybrids evaluated in the Highlands, Subtropical and Tropical MasAgro seed evaluation networks in Mexico, 2011–2019. Source: [33].
(DOCX)

S1 Fig Sampled MasAgro SMEs product portfolio composition of maize varieties by product type, 2011–2019 (n = 31). Source: [34,41,42].
(DOCX)

S2 Fig Sampled MasAgro SMEs composition of maize seed sales by product type, 2011–2019 (n = 31). Source: Source: [34,41,42].
(DOCX)

S3 Fig Sampled MasAgro SMEs product portfolio composition of maize varieties by mega-environment, 2011–2019 (n = 31). Source: [34,41,42].
(DOCX)

S4 Fig Sampled MasAgro SMEs composition of maize seed sales by mega-environment, 2011–2019 (n = 31). Source: [34,41,42].
(DOCX)

S5 Fig Sampled MasAgro SMEs product portfolio composition of maize varieties by product type and company size, 2011–2019 (n = 31). Source: [34,41,42].
(DOCX)

S6 Fig Sampled MasAgro SMEs composition of maize seed sales by product type and company size, 2011–2019 (n = 31). Source: [34,41,42].
(DOCX)

## References

[1] Evenson R, Gollin D. Crop variety improvement and its effect on productivity: the impact of international agricultural research. Wallingford: CABI Publishing; 2003. doi: 10.1079/9780851995496.0447

[2] Byerlee D, Dubin HJ. Crop improvement in the CGIAR as a global success story of open access and international collaboration. Int J Commons. 2009;4(1):452. doi: 10.18352/ijc.147

[3] Pray CE. Public‐private sector linkages in research and development: biotechnology and the Seed Industry in Brazil, China and India. Am J Agric Econ. 2001;83(3):742–7. doi: 10.1111/0002-9092.00201

[4] Naseem A, Spielman DJ, Omamo SW. Private‐sector investment in R&D: a review of policy options to promote its growth in developing‐country agriculture. Agribusiness. 2010;26(1):143–73. doi: 10.1002/agr.20221

[5] Morris M, Edmeades G, Pehu E. The global need for plant breeding capacity: what roles for the public and private sectors? HortSci. 2006;41(1):30–9. doi: 10.21273/hortsci.41.1.30

[6] Fuglie K. The growing role of the private sector in agricultural research and development world-wide. Glob Food Sec. 2016;10:29–38. doi: 10.1016/j.gfs.2016.07.005

[7] Srinivasan CS. Concentration in ownership of plant variety rights: some implications for developing countries. Food Policy. 2003;28(5–6):519–46. doi: 10.1016/j.foodpol.2003.10.003

[8] Kapur A. IPR laws to protect innovation not restrict crop breeding - a rational approach. J Intellect Prop Rights. 2011;16:117–23.

[9] Galluzzi G, Halewood M, Noriega IL, Vernooy R. Twenty-five years of international exchanges of plant genetic resources facilitated by the CGIAR genebanks: a case study on global interdependence. Biodivers Conserv. 2016;25(8):1421–46. doi: 10.1007/s10531-016-1109-7

[10] Hall A, Bockett G, Taylor S, Sivamohan MVK, Clark N. Why research partnerships really matter: innovation theory, institutional arrangements and implications for developing new technology for the poor. World Dev. 2001;29(5):783–97. doi: 10.1016/s0305-750x(01)00004-3

[11] Gowda CL, Reddy BVS, Saxena K. ICRISAT collaboration with the seed industry in Asia. Seoul (South Korea): International Crops Research Institute for the Semi-Arid Tropics (ICRISAT); 2004.

[12] Byerlee D, Fischer K. Accessing Modern Science: Policy and Institutional Options for Agricultural Biotechnology in Developing Countries. World Dev. 2002;30(6):931–48. doi: 10.1016/s0305-750x(02)00013-x

[13] Ervin D, Lomax T, Buccola S, Kim K, Minor E, Yang H. University-industry relationships: framing the issues for academic research in agricultural biotechnology, proceedings from an expert workshop Nov 19-20, 2002. North Carolina: Portland State University; 2003. https://www.iprsonline.org/research/UIR.pdf

[14] Syngenta Foundation for Sustainable Agriculture [Internet]. Basel: Public-private partnerships: A guidance framework. [cited 2023 Dec 23]. Available from: https://www.syngentafoundation.org/public-private-partnerships-guidance-framework

[15] Ferroni M, Castle P. Public-Private Partnerships and Sustainable Agricultural Development. Sustainability. 2011;3(7):1064–73. doi: 10.3390/su3071064

[16] Hartwich F, Tola J, Engler A, González C, Ghezan G, Vázquez-Alvarado JMP, et al. Building public-private partnerships for agricultural innovation. Washington (DC): International Food Policy Research Institute (IFPRI); 2008. doi: 10.2499/9780896297715fsp4

[17] Spielman DJ, Hartwich F, von Grebmer K. Sharing science, building bridges, and enhancing impact, public-private partnerships in the CGIAR. Washington (DC): International Food Policy Research Institute (IFPRI); 2007.

[18] Smyth SJ, Webb SR, Phillips PWB. The role of public-private partnerships in improving global food security. Glob Food Sec. 2021;31:100588. doi: 10.1016/j.gfs.2021.100588

[19] Tefera T, Mugo S, Beyene Y. Developing and deploying insect resistant maize varieties to reduce pre-and post-harvest food losses in Africa. Food Sec. 2016;8(1):211–20. doi: 10.1007/s12571-015-0537-7

[20] Edmeades GO. Progress in achieving and delivering drought tolerance in maize - an update. Ithaca (NY): International Service for the Acquisition of Agri-biotech Applications (ISAAA); 2013.

[21] Simtowe F, Marenya P, Amondo E, Worku M, Rahut DB, Erenstein O. Heterogeneous seed access and information exposure: implications for the adoption of drought-tolerant maize varieties in Uganda. Agric Food Econ. 2019;7(1). doi: 10.1186/s40100-019-0135-7

[22] Syngenta Foundation for Sustainable Agriculture [Internet]. Basel: AAA Maize. [cited 2023 Dec 23]. Available from: https://www.syngentafoundation.org/aaa-maize

[23] Ingram GM, Johnson AE, Moser H. USAID’s public-private partnerships: A data picture and review of business engagement. Washington (DC): Global Economy and Development at Brookings; 2016.

[24] Brooks SH. Philanthrocapitalism, ‘propoor’ agricultural biotechnology and development. In: Morvaridi B, editor. New philanthropy and social justice: Debating the conceptual and policy discourse. Bristol: Bristol University Press, Policy Press; 2015. pp. 101–14.

[25] Donovan J, Rutsaert P, Domínguez C, Peña M. Capacities of local maize seed enterprises in Mexico: Implications for seed systems development. Food Secur. 2022. doi: 10.1007/s12571-021-01247-8

[26] Byerlee D, Traxler G. National and international wheat improvement research in the post‐green revolution period: evolution and impacts. Am J Agric Econ. 1995;77(2):268–78. doi: 10.2307/1243537

[27] Atlin GN, Cairns JE, Das B. Rapid breeding and varietal replacement are critical to adaptation of cropping systems in the developing world to climate change. Global Food Security. 2017;12:31–7. doi: 10.1016/j.gfs.2017.01.008 28580238 PMC5439485

[28] Andorf C, Beavis WD, Hufford M, Smith S, Suza WP, Wang K, et al. Technological advances in maize breeding: past, present and future. Theor Appl Genet. 2019;132(3):817–49. doi: 10.1007/s00122-019-03306-3 30798332

[29] Kotler P, Keller LK. Marketing management. 14th ed. New Jersey: Prentice Hall; 2012.

[30] CIMMYT [Internet]. Texcoco: MasAgro Maize. [cited 2020 Jul 22]. Available from: https://www.cimmyt.org/projects/masagro-maize/

[31] Walker TS, Alwang J. Crop improvement, adoption, and impact of improved varieties in food crops in Sub-Saharan Africa. Walker TS, Alwang J, editors. Wallingford: CGIAR-CABI; 2015.

[32] Spielman DJ, Kennedy A. Towards better metrics and policymaking for seed system development: Insights from Asia’s seed industry. Agric Syst. 2016;147:111–22. doi: 10.1016/j.agsy.2016.05.015 27594738 PMC4952526

[33] Domínguez C, Donnet L, Silva-Hinojosa A, López-Becerril DI, Burgueño J. MasAgro maize seed evaluation network yield data, 2011-2019 [dataset]. CIMMYT Research Data & Software Repository Network. 2024. Available from https://hdl.handle.net/11529/10549157

[34] Domínguez C, Donnet L, Silva-Hinojosa A, López-Becerril DI, Burgueño J. MasAgro seed marketing survey, 2013-2019 [dataset]. CIMMYT Research Data and Software Repository Network. 2024. Available from https://hdl.handle.net/11529/10549158

[35] SNICS [dataset]. Catalogo Nacional de Variedades Vegetales en linea, última actualización 7 de Septiembre del 2020. Ciudad de México CDMX: Servicio Nacional de Inspección y Certificación de Semillas (SNICS); 2020 [cited 2020 Sep 7]. Available from: https://datastudio.google.com/reporting/5b7206ba-e190-48fe-9696-73523bfccf58/page/itBWB

[36] Torres Flores JL, García BM, Prasanna BM, Alvarado G, San Vicente FM, Crossa J. Grain yield and stability of White Early Maize Hybrids in the Highland Valleys of Mexico. Crop Sci. 2017;57(6):3002–15. doi: 10.2135/cropsci2017.03.0145

[37] Brennan JP, Byerlee D. The rate of crop varietal replacement on farms: Measures and empirical results for wheat. Plant Var Seeds. 1991;4:99–106.

[38] Hölzl W. Is the R&D behaviour of fast-growing SMEs different? Evidence from CIS III data for 16 countries. Small Bus Econ. 2009;33(1):59–75. doi: 10.1007/s11187-009-9182-x

[39] Klingebiel R, Rammer C. Resource allocation strategy for innovation portfolio management. Strat Mgmt J. 2013;35(2):246–68. doi: 10.1002/smj.2107

[40] Grimpe C, Kaiser U. Balancing internal and external knowledge acquisition: The gains and pains from R&D outsourcing. J Manag Stud. 2010;47(8).

[41] Domínguez C, Donnet L, Silva-Hinojosa A, López-Becerril DI, Burgueño J. CIMMYT germplasm impact survey, 2015 [dataset]. CIMMYT Research Data and Software Repository Network. 2024. Available from https://hdl.handle.net/11529/10549158

[42] Domínguez C, Donnet L, Silva-Hinojosa A, López-Becerril DI, Burgueño J. MasAgro product portfolio survey, 2020 [dataset]. 2024. CIMMYT Research Data and Software Repository Network. Available from https://hdl.handle.net/11529/10549158

[43] INEGI [dataset]. Sistema de Clasificación Industrial de América del Norte (SCIAN). Ciudad de México [CDMX]: Instituto Nacional de Estadística y Geografía (INEGI). 2023 [cited 2023 Apr 25]. Available from: https://www.inegi.org.mx/scian/

[44] SNICS [dataset]. 2011-2019, Existencias de Semillas. Ciudad de México [CDMX]: Servicio Nacional de Inspección y Certificación de Semillas (SNICS); 2019.

[45] Domínguez C, Donnet L, Silva-Hinojosa A, López-Becerril DI, Burgueño J. Mexico’s maize seed production and sales dataset, 2011-2019 [dataset]. CIMMYT Research Data and Software Repository Network. 2024. Available from: https://hdl.handle.net/11529/10549158

[46] Rhoades SA. Market share inequality, the HHI, and other measures of the firm-composition of a market. Rev Ind Organ. 1995;10:657–74.

[47] Tanusondjaja A, Dunn S, Miari C. Examining manufacturer concentration metrics in consumer packaged goods. Int J Mark Res. 2020;63(4):471–93. doi: 10.1177/1470785320903978

[48] Ginevičius R, Čirba S. Additive measurement of market concentration. J Bus Econ Manag. 2009;10(3):191–8. doi: 10.3846/1611-1699.2009.10.191-198

[49] Sayaka B. Market structure of the corn seed industry in East Java. J Agro Ekon. 2006;24(2):133–56.

[50] SIAP [dataset]. Datos abiertos estadística de producción agrícola. Ciudad de México [CDMX]: Servicio de Información Agroalimentaria y Pesquera (SIAP). 2020 [cited 2020 Aug 6]. Available from: http://infosiap.siap.gob.mx/gobmx/datosAbiertos.php

[51] FAOSTAT Production [Internet]. Rome [LAZ]: Food and Agriculture Organization of the United Nations (FAO). 2020. [cited 2020 Nov 11]. Available from: http://www.fao.org/faostat/en/#data

[52] SIAP [dataset]. Boletín balanza disponibilidad-consumo. Ciudad de México [CDMX]: Servicio de Información Agroalimentaria y Pesquera (SIAP); 2020 [cited 2020 Aug 6]. Available from: https://www.gob.mx/siap/documentos/balanzas-disponibilidad-consumo-de-productos-agropecuarios-seleccionados-104471

[53] FAOSTAT Trade [Internet]. Rome [LAZ]: Food and Agriculture Organization of the United Nations (FAO). 2022. [cited 2022 Feb 27]. Available from: https://www.fao.org/faostat/en/#data

[54] SIAP [dataset]. Tecnificación. Ciudad de México [CDMX]: Servicio de Información Agroalimentaria y Pesquera (SIAP); 2016 [cited 2020 Aug 6]. Available from: https://www.gob.mx/siap/documentos/tecnificacion

[55] INEGI. Marco Geoestadístico. Ciudad de México [CDMX]: Instituto Nacional de Estadística y Geografía (INEGI); 2021. Available from: https://www.inegi.org.mx/temas/mg/

[56] Ayala-Garay OJ, García-de los Santos G, Ayala-Garay AV, Schwentesius-Rindermann R. Influencia de aspectos normativos en el desarrollo de la investigación, la enseñanza y la producción de semillas, el ejemplo del Colegio de Postgraduados. In: Schwentesius-Rindermann R, Gómez-Cruz MA, Ayala-Garay AV, editors. Memorias del Foro Nacional Agenda del Desarrollo 2006-2020: Políticas de Desarrollo Agropecuario, Forestal y Pesquero. Texcoco: CIESTAAM-Universidad Autónoma Chapingo; 2006. pp. 149–63.

[57] Ley sobre producción, certificación y comercio de semillas 1961 (Apr 14, 1961). Available from: http://dof.gob.mx/index_113.php?year=1961&month=04&day=14

[58] Morris ML. Maize seed industries in developing countries. Texcoco: CIMMYT; 1998.

[59] Echeverría RG. Public and private investments in maize research in Mexico and Guatemala. Mexico, DF: CIMMYT; 1990.

[60] Secretaría de Economía [dataset]. Sistema de Consulta de Información Estadística por País. Ciudad de México [CDMX]: Secretaría de Economía; 2019 [cited 2019 May 19]. Available from: https://www.gob.mx/se/acciones-y-programas/comercio-exterior-informacion-comercial?state=published. Spanish.

[61] Espinosa CA, Tadeo RM, Turrent FA. Concentración de la oferta de semillas mejoradas de maíz. La Jornada. 2010. Available from: https://www.jornada.com.mx/2010/03/13/oferta.html. Spanish.

[62] Witcombe JR, Packwood AJ, Raj AGB, Virk DS. The extent and rate of adoption of modern cultivars in India. In: Witcombe JR, Farrington D, editors. Seeds of Choice: Making the most of new varieties for small farmers. New Delhi and London: Oxford IBH Intermediate Technology Publications; 1998. pp. 53–68.

[63] Nairobi: Welcome to QBS; [cited 2022 Mar 18]. Available from: https://qualibasicseed.com/

[64] International Service for the Acquisition of Agri-biotech Applications. AATF establishes first early generation seed production entity in Sub-Saharan Africa. ISSAA Knowledge Center Biotech Updates [Internet]. 2017 [cited 2022 Mar 18]. Available from: https://www.isaaa.org/kc/cropbiotechupdate/article/default.asp?ID=15139

[65] Waithaka M, Mugoya M, Mabaya E, Tihanyi K. Decentralized seed services in Africa: An assessment of Tanzania and Uganda. Bonn, Germany: Center for Development Research, University of Bonn; 2021.

[66] Donnet ML, López Becerril ID, Domínguez Méndez C, Arista Cortés J. Análisis de la estructura del sector y la asociación público-privada de semillas de maíz en México. Agron Mesoam. 2020:367–83. doi: 10.15517/am.v31i2.34894

[67] Erenstein O, Kassie GT. Seeding eastern Africa’s maize revolution in the post-structural adjustment era: a review and comparative analysis of the formal maize seed sector. Int Food Agribus Manag Rev. 2018;21(1):39–52. doi: 10.22434/ifamr2016.0086

[68] Kassie GT, Erenstein O, Mwangi W, MacRobert J, Setimela P, Shiferaw B. Political and economic features of the maize seed industry in southern Africa. Agrekon. 2013;52(2):104–27. doi: 10.1080/03031853.2013.798067

[69] Langyintuo AS, Mwangi W, Diallo AO, MacRobert J, Dixon J, Bänziger M. Challenges of the maize seed industry in eastern and southern Africa: a compelling case for private–public intervention to promote growth. Food Policy. 2010;35(4):323–31. doi: 10.1016/j.foodpol.2010.01.005

[70] Camacho-Villa TC, Almekinders C, Hellin J, Martinez-Cruz TE, Rendon-Medel R, Guevara-Hernández F, et al. The evolution of the MasAgro hubs: responsiveness and serendipity as drivers of agricultural innovation in a dynamic and heterogeneous context. J Agric Educ Exten. 2016;22(5):455–70. doi: 10.1080/1389224x.2016.1227091

[71] Domínguez C, Donovan J, Srinivasan CS, Zanello G, Peña M. In-store seed purchasing decisions, implications for scaling hybrid maize seed sales through agro-dealers. Research Square: rs.3.rs-2346961/v1 [Preprint]. 2022 [cited 6 Dec 2022]. Available from: https://www.researchsquare.com/article/rs-2346961/v1

[72] Heiman A, Hildebrandt L. Marketing as a Risk Management Mechanism with Applications in Agriculture, Resources, and Food Management. Ann Rev Resour Econ. 2018;10(1):253–77. doi: 10.1146/annurev-resource-100517-023047

[73] Heiman A, Ferguson J, Zilberman D. Marketing and Technology Adoption and Diffusion. Appl Econ Perspect Pol. 2020;42(1):21–30. doi: 10.1002/aepp.13005

[74] Louwaars NP, de Boef WS. Integrated Seed Sector Development in Africa: A Conceptual Framework for Creating Coherence Between Practices, Programs, and Policies. J Crop Improv. 2012;26(1):39–59. doi: 10.1080/15427528.2011.611277

