# Supplementary material for: Public-private partnerships for seed industry development in developing countries: Lessons from MasAgro maize in Mexico
Source: PLoS One. 2025 Aug 6;20(8):e0328872. doi: 10.1371/journal.pone.0328872 (PMC12327655; doi:10.1371/journal.pone.0328872)
Supplement: S1 Appendix — (DOCX) [file pone.0328872.s001.docx]

# **S1 Appendix. Examples of public-private partnerships (PPPs) in plant breeding**

| **Project name – project span** | **Country/region** | **Crops** | **Major partners** | **Project objectives** | **Main results/outputs** | **Reference** |
| --- | --- | --- | --- | --- | --- | --- |
| Virus-resistant sweet potato (1991-2007). | Kenya, Egypt, Indonesia, Costa Rica. | Sweetpotato | USAID, MSU, KARI, ISAAA, CIP, DDPSC, ARC-Roodeplaat VOPI South Africa, Monsanto. | Development of transgenic sweet potato  Technology and technology transfer to developing countries. | • Successful GMO transformation, but varieties showed low-level virus resistance.  • Enhanced research biotechnology capacities.  • Facilitated operationalization of Kenyan Biosafety system for GM testing and approval.  • Introduction of transgenic sweet potato permit in Kenya issued in 1999.  • Transfer of the recombinant sweetpotato technology from Monsanto to KARI in 2000. | Odame et al. 2002; Wambugu, 2003. |
| Insect-Resistant Maize for Africa IRMA (1999-2003). | Kenya | Maize | CIMMYT, KARI, Syngenta Foundation, Monsanto, Wakala Seeds. | Development and delivery of maize varieties resistant to stem borers and postharvest pests. | • Development of source lines of the key Bt genes Cry1Ab and Cry1Ba. • Release of 13 stem borer-resistant non-GM maize varieties (3 OPVs and 10 hybrids) and 4 storage pest-resistant hybrids in Kenya between 2006 and 2011. • 3 insect-resistant (IR) hybrids (KH 414-1 SBR, 414-4 SBR) and the OPV Pamuka commercialised in Kenya by Monsanto, Wakala Seeds and KARI Seed. | Mugo et al. 2005; Mabeya and Ezezika, 2012; Tefera et al. 2016. |
| The sorghum, pearl millet and pigeonpea Hybrid Parents Research Consortia (2000-2017). | India, Egypt, Indonesia and Thailand. | Sorghum, pearl millet and pigeonpea. | ICRISAT, private companies (MAHYCO, JK Agri Genetics, Proagro Seed Company/Bayer, Vikki’s Agro-Tech Ltd, Ganga Kaver i Seeds, Biogene, Monsanto). | Develop sorghum, pearl millet and pigeonpea hybrid cultivars adapted and resistant to major biotic and abiotic stresses. Promote private seed sector development. | • 50 private companies were members of one or more consortia by 2008. • 15 sorghum hybrids developed by affiliated companies using ICRISAT-bred materials. • 103 pearl millet hybrids developed between 2000 and 2010. • 8 pigeonpea hybrids directly released by the private sector using ICRISAT parental lines. • 25 consortium companies marketed 82 pearl millet hybrids using ICRISAT-derived breeding materials by 2006. | Gowda et al. 2006; Mula et al. 2007; Ventaka et al. 2018. |
| Striga-Resistant Maize Project - STRIGAWAY (2006-2014). | Kenya, Malawi, Uganda, Tanzania. | Maize, sorghum, millet and rice. | CIMMYT, AATF, KARI, NARs of participating countries (KALRO, DARS, NARO, TARI), BASF, regional seed companies. | Introduce maize, sorghum, millet and rice varieties resistant to the StrigAway herbicide (BASF®). | • STRIGAWAY® technology developed • Insect-resistant (IR) maize hybrid Ua Kayongo (Striga Killer) commercially launched in Kenya in 2006. • Over 100t of certified seed of the hybrid Ua Kayongo (Striga killer) produced by WSC for large scale testing in 2007. • 10t of IR maize seed distributed by AATF in 2007. • 6 early OPVs, 5 late OPVs and 2 hybrids allocated to seed companies and NARS for registration as new varieties and subsequent commercialization. | Spielman et al. 2007; Odame and Muangue, 2011. |
| HarvestPlus (2004 to date). | Africa, Asia, Latin America and The Caribbean | Wheat, maize, rice, beans, pearl millet, cassava, potato, sweetpotato. | ETH Zurich, ICRISAT, CIMMYT, CIP, IRRI, The World Bank, GAIN, DFID, the Rockefeller Foundation, Syngenta (Greenovation, Zeneca), BMBF, NGOs, NARS, PhilRice. | Develop new, more nutritious varieties of staple food crops that provide higher amounts of vitamin A, iron, or zinc - and reach 1 billion people with biofortified foods by 2030. | • Biofortified crops (vitamin A orange sweetpotato, iron beans, iron pearl millet, vitamin A yellow cassava, vitamin A orange maize, zinc rice, and zinc wheat) released +30 countries, tested and grown in +40 countries.  • +33 million people are growing and eating biofortified foods.  • +290 biofortified varieties of 12 staple food crops have been released, and hundreds more are being tested in +60 countries around the world." | Lalani et al. 2019; Bouis and Saltzman, 2017; Brooks, 2015. |
| Drought-Tolerant Maize for Africa DTMA - (2006-2015) and Drought Tolerant Maize for Africa Seed Scaling - DTMASS (2015-2020). | Sub-Saharan Africa | Maize | CIMMYT, IITA, USAID, DFID, BMGF, Howard G. Buffett Foundation, CSIR (Kenya), NARs (ARCN, KALRO, NARO, EIAR, ZARI, DRSS) small and medium seed companies. | Develop and disseminate drought-tolerant (DT) maize hybrids in 13 countries of Sub-Saharan Africa; Scaling up and out drought tolerant, stress-resilient and high-yielding maize hybrids. | • 233 varieties (including about 200 distinct DT maize varieties) released across target countries as of 2016. • African NARs providing 300t of breeder seed annually to community-based seed production schemes. • Over 33,000t (1,650,000 sixty thousand seeds bags) of seed distributed to farmers in participating SSA. | Edmeades, 2013; Martey et al. 2020. |
| AGRA Program for Africa’s Seed Systems - PASS (2006- to date). | Africa: eventually grew to include 18 countries | Staples | USAID, The Rockefeller Foundation, BMGF, Howard G. Buffett Foundation. | Develop and release new crop varieties; Strengthen Africa's breeding and seed sector capacities; Build agro-dealer networks to sell improved seed and other inputs to local, smallholder farmers. | • 114 African seed companies producing over 128,000 t of certified seed annually. • Public crop breeding teams developed and released 600 new crop varieties. Over 400 of these were at some stage of commercialization. **•** About 25,000 agro-dealers trained and certified for operation as private input suppliers in 18 countries across Africa. • A total of 404,000 million t of seed and over 1.1 million t of fertilizer sold to farmers through agro-dealers trained by 2018. | AGRA, 2017. |
| Water-Efficient Maize for Africa WEMA (2008-2018). | Eastern and Southern Africa | Maize | CIMMYT, AATF, BMGF, Howard G. Buffett Foundation, NARs of participant countries (KALRO, TARI, ARC, IIAM, NARO), Monsanto, BASF. | Development of water-efficient and insect resistant (IR) maize hybrids for their performance to drought conditions in Africa. | • Successful confined field trials for GM maize varieties in Kenya, Uganda and South Africa (2008-2013). • First WEMA non-GM drought tolerant maize hybrids scheduled to be released in 2013.  • Bacillus thuringiensis (Bt) insect resistance traits (MON87460 and MON810) in confined field trial testing stage in 2013 in Uganda and Kenya. • Bt IR and DT WEMA GM hybrids scheduled for release by 2016/2017. | Odame and Muangue, 2011; Edmeades, 2013; Thomson, et al. 2020. |
| Affordable, Accessible Asian Drought Tolerant Maize Project - AAA DT Maize (2010-2016). | Southeast Asia (India), Indonesia, Philippines and Vietnam | Maize | CIMMYT, Syngenta, Syngenta Foundation, NARs of Indonesia, Philippines and Vietnam. | Develop drought-tolerant, low-cost maize hybrids for smallholder farmers in low-rainfall drought-prone areas of South Asia. | • Hybrid TA5084 first sold during the 2018 Kharif (monsoon) season and promoted by around ten Indian seed partners (either small companies or NGOs) in Madhya Pradesh, Rajasthan and Gujarat states of India. • Local seed partners sold 18 t of AAA maize in 2018, 50 t in 2019 and 120 tons in 2020. • Some 8k farmers in central India planted the seeds on about 6000 ha. • Local seed companies and NGOs selling 4kg and 1kg bags under the AAA brand. | CIMMYT, n.d. Syngenta Foundation, 2021. |
| International Maize Improvement Consortium IMIC-Asia - (2010- to date) and IMIC-Africa (2018 to date). | South and Southeast Asia, mainly India Africa: Ethiopia, Kenya, Zimbabwe | Maize | Asia: CIMMYT, GIZ/GMBH, local seed companies  Africa: CIMMYT, NARs of participating countries (EIAR, KALRO, DRSS), private seed companies. | Develop improved early and advanced maize lines; evaluate pre-release CIMMYT-bred hybrids and hybrid combinations of partners; strengthen public and private maize breeding and seed sector capacities. | • 43 affiliated members from the public and the private sectors by 2014. • 317 CIMMYT-bred hybrids distributed to members for in-house evaluation and shortlist the best five hybrids in 2014. • Deployment of hybrids once registered was expected to be made by private companies. • IMIC-Africa launched in 2018 | Sadananda et al. 2014. |
| Improved Maize for African Soils (IMAS) - 2010-2017. | Eastern and Southern Africa | Maize | CIMMYT, USAID, KALRO, South African ARC, KARI, BMGF, DuPont-Pioneer. | Development of high yielding maize varieties with improved nitrogen use efficiency (NUE) | • The largest tropical N stress phenotyping network in the world, with more than 120,000 N-depleted research plots at 16 experimental stations in seven countries established. • 10 NUE lines used as donor parents in more than 600 pedigree starts, crossed with Africa adapted germplasm and being advanced through pedigree selection and doubled haploid technology. | Semagn et al. 2015. |
| Stress Tolerant Maize for Africa - STMA (2016-2020). | Eastern, Southern and West Africa. | Maize | CIMMYT, USAID, BMGF, NARs of participating countries (EIAR, KALRO, TARI, NARO, DARS, ARC, ZARI, DRSS, etc.), private companies. | Develop improved maize varieties with resistance and tolerance to drought, low soil fertility, heat, maize lethal necrosis and pests affecting maize production. | • 15 varieties released in 2016, 17 in 2017 and 14 in 2018. • 36,622t of certified seed produced in 2016, 39,917t in 2017, 54,565t in 2018 and >85,000 in 2019. | Simtowe, 2019. |
| TELA Maize Project (2018-2024). | Eastern and Southern Africa | Maize | CIMMYT, AATF, ARCN, ARC, IIAM, TARI, NARO, KALRO, EIAR, USAID, BMGF, Howard G. Buffett Foundation, Bayer. | Commercialization of transgenic drought-tolerant and insect-resistant maize varieties in sub-Saharan Africa. | • Bacillus thuringiensis (Bt) stem borer and fall armyworm resistance traits (MON810 and MON89034) in confined field trial testing stage in Ethiopia, Kenya, Mozambique, South Africa, Tanzania and Uganda.  • 5 TELA® hybrids commercialized in South Africa since 2016." | Thomsom et al. 2020; AATF, n.d. |

# **Partners:** United States Agency for International Development (USAID); Michigan State University (MSU); Kenyan Agricultural Research Institute (KARI); International Service for the Acquisition of Agri-biotech Applications (ISAAA); International Potato Center (CIP); Donald Danforth Plant Sciences Center (DDPSC); Agricultural Research Council Roodeplaat, Vegetable and Ornamental Plant Institute (ARC-Roodeplaat VOPI South Africa); International Maize and Wheat Improvement Center (CIMMYT); International Crops Research Institute for the Semi-Arid Tropics (ICRISAT); African Agricultural Technology Foundation (AATF); Kenia Agricultural & Livestock Research Organization (KALRO); Malawi’s Department of Agricultural Research Services (DARS); Uganda's National Agricultural Research Organization (NARO); Tanzanian Institute of Agricultural Research (TARI); Agricultural Research Council of South Africa (ARC); Mozambique Institute of Agricultural Research (IIAM); Agricultural Research Council of Nigeria (ARCN); Ethiopian Institute of Agricultural Research (EIAR); Zambian Agricultural Research Institute (ZARI); Zimbabwe’s Department of Research and Specialist Services (DRSS); International Institute for Tropical Agriculture (IITA); UK Department for International Development (DFID); Bill and Melinda Gates Foundation (BMGF); Deutsche Gesellschaft Fuer Internationale Zusammenarbeit (GIZ/GMBH); Global Alliance for Improved Nutrition (GAIN); Philippine Rice Research Institute (PhilRice).

# **References**

African Agricultural Technology Foundation. TELA Maize Technology, FAQs: All you need to know. TELA Maize Project. Sponsored by the Bill & Melinda Gates Foundation, Howard G. Buffet Foundation and the United States Agency for International Development (USAID). Available at https://www.aatf-africa.org/wp-content/uploads/2021/02/TELA-Project-FAQ.pdf

Alliance for a Green Revolution in Africa. Seeding an African Green Revolution: The Pass Journey. Nairobi, Kenya: Alliance for a Green Revolution in Africa (AGRA); 2017. 212p.

Bouis HE, Saltzman A. Improving nutrition through biofortification: A review of evidence from HarvestPlus, 2003 through 2016. Glob Food Sec. 2017; 12: 49-58. doi: 10.1016/j.gfs.2017.01.009

Brooks SH. Philanthrocapitalism, ‘propoor’ agricultural biotechnology and development. In: Morvaridi B, editor. New philanthropy and social justice: Debating the conceptual and policy discourse. Bristol: Bristol University Press, Policy Press; 2015. p. 101-114.

CIMMYT [Internet]. Texcoco: Affordable, Accessible Asian (AAA) Drought Tolerant Maize Project [cited 2022 Aug 21]. Projects; [about 2 screens]. Available from https://www.cimmyt.org/projects/affordable-accessible-asian-aaa-drought-tolerant-maize-project/

Edmeades G.O. Progress in achieving and delivering drought tolerance in Maize - An Update. Ithaca (NY): International Service for the Acquisition of Agri-biotech Applications (ISAAA); 2013. ISAAA Brief 44.

Gowda CLL, Rai KN, Reddy BVS, Saxena KB, editors. Hybrid parents research at ICRISAT. Andhra Pradesh, India: International Crops Research Institute for the Semi-Arid Tropics (ICRISAT); 2006. 212 p.

Lalani B, Bechoff A, Bennett B. Which choice of delivery model(s) works best to deliver fortified foods? Nutrients. 2019; 11: 1594. doi: 10.3390/nu11071594

Mabeya J, Ezezika OC. Unfulfilled farmer expectations: The case of the Insect Resistant Maize for Africa (IRMA) project in Kenya. Agric Food Secur*.* 2012; 1(1): S6. http://www.agricultureandfoodsecurity.com/content/1/S1/S6

Martey E, Prince M, Etwirea J, Kuwornuc KM. Economic impacts of smallholder farmers’ adoption of drought-tolerant maize varieties. Land Use Policy*.* 2020; 94: 104524. doi: 10.1016/j.landusepol.2020.104524

Mugo S, de Groote H, Bergvinson D, Mulaa M, Songa J, Gichuki S. Developing Bt maize for resource-poor farmers – Recent advances in the IRMA project. Afr J Biotechnol. 2005; 4(13): 1490-1504. http://www.academicjournals.org/AJB

Mula RP, Rai KN, Kulkarni VN, Singh AK. Public-private partnership and impact of ICRISAT’s pearl millet hybrid parents research. J of SAT Agric Res. 2007; 5(1).

Odame H, Kameri-Mbote P, Wafula D. Innovation and policy process: Case of transgenic sweet potato in Kenya. Econ Polit Wkly. 2002; 37(27): 2770-2777. https://www.jstor.org/stable/4412332

Odame H, Muange E. Can agro-dealers deliver the green revolution in Kenya? IDS Bull. 2011*;* 42: 78–89. doi: 10.1111/j.1759-5436.2011.00238.x

Sadananda A, Vivek BS. Zaidi PH. International Maize Improvement Consortium (IMIC) in Asia: Partnership with seed partners for client-oriented product development and delivery. In: Prasanna BM, Vivek BS, Sadananda AR, Jeffers D, Zaidi PH, Boeber C, et al., editors. 12th Asian Maize Conference and Expert Consultation on Maize for Food, Feed, Nutrition and Environmental Security; 2014 October 30 – November 1; Bangkok, Thailand. Mexico DF: International Maize and Wheat Improvement Center (CIMMYT). 2014. p. 231-237.

Semagn K, Beyene Y, Babu R, Nair S, Gowda M, Das B, et al. Quantitative trait loci mapping and molecular breeding for developing stress resilient maize for sub-Saharan Africa. Crop Sci. 2015; 55: 1449–1459. doi: 10.2135/cropsci2014.09.0646

Simtowe F, Muricho DN, Mbando F, Makumbi D, McDonald J. How scalable are stress tolerant maize varieties? An examination of knowledge, seed access and affordability heterogeneity effect in Tanzania. 6th African Conference of Agricultural Economists; 2019 Sep 23-26. Abuja, Nigeria.

Spielman, D.J., Hartwich, F. & von Grebmer, K. (2007). Sharing science, building bridges, and enhancing impact, public-private partnerships in the CGIAR. Washington (DC): International Food Policy Research Institute (IFPRI); 2007. IFPRI Discussion Paper 00708.

Syngenta Foundation for Sustainable Agriculture [Internet]. Basel: AAA Maize: [cited 2023 Dec 23]. [About 2 screens]. Available from https://www.syngentafoundation.org/aaa-maize

Tefera T, Mugo S, Beyene Y. Developing and deploying insect resistant maize varieties to reduce pre-and post-harvest food losses in Africa. Food Secur. 2016; 8: 211–220. https://doi.org/10.1007/s12571-015-0537-7

Thomson JA, Oikeh SO, Sithole-Niang I, Tripathi L. Advanced genetic technologies for improving plant production. In: Sikora RA, Terry ER, Vlek PLG, Chitja J, editors. Transforming agriculture in Southern Africa, constraints, technologies, policies and processes. London and New York: Routledge, Taylor & Francis; 2020. p. 162-169.

Venkata RN, Rao KPC, Gupta SK, Mazvimavi K, Kumara CD, Nagaraj N, et al. Impact of ICRISAT Pearl Millet Hybrid Parents Research Consortium (PMHPRC) on the livelihoods of farmers in India. Patancheru, India: International Crops Research Institute for the Semi-Arid Tropics (ICRISAT); 2018. Research Report 75.

Wambugu FM. Development and transfer of genetically modified virus-resistant sweet potato for subsistence farmers in Kenya. Nutr Rev. 2003; 61(6): S110 –S113.
