# Supplementary material for: Public-private partnerships for seed industry development in developing countries: Lessons from MasAgro maize in Mexico
Source: PLoS One. 2025 Aug 6;20(8):e0328872. doi: 10.1371/journal.pone.0328872 (PMC12327655; doi:10.1371/journal.pone.0328872)
Supplement: S1 Table — Source: [33]. (DOCX) [file pone.0328872.s002.docx]

# **S1 Table. Grain yield (GY) means, GY differences, significance levels and standard errors of MasAgro, multinational, private national and public hybrids evaluated in the Highlands, Subtropical and Tropical MasAgro seed evaluation networks in Mexico, 2011-2019.**

| Mega-environment/colour | GY | MasAgro | | Multinational | | Private national | | Public | |
| --- | --- | --- | --- | --- | --- | --- | --- | --- | --- |
|  |  | Diff | SE | Diff | SE | Diff | SE | Diff | SE |
| Subtropical white |  |  |  |  |  |  |  |  |  |
| MasAgro | 8.18 | -- | -- |  |  |  |  |  |  |
| Multinational | 8.74 | 0.56** | 0.25 | -- | -- |  |  |  |  |
| Private national | 8.63 | 0.45* | 0.25 | -0.11 | 0.22 | -- | -- |  |  |
| Public | 7.83 | -0.35 | 0.26 | -0.91*** | 0.23 | -0.80*** | 0.22 | -- | -- |
| Subtropical yellow |  |  |  |  |  |  |  |  |  |
| MasAgro | 7.99 | -- | -- |  |  |  |  |  |  |
| Multinational | 8.81 | 0.83** | 0.32 | -- | -- |  |  |  |  |
| Private national | 8.13 | 0.14 | 0.23 | -0.69** | 0.28 | -- | -- |  |  |
| Public | 7.04 | -0.95** | 0.39 | -1.78*** | 0.43 | -1.09*** | 0.37 | -- | -- |
| Highlands white |  |  |  |  |  |  |  |  |  |
| MasAgro | 7.01 | -- | -- |  |  |  |  |  |  |
| Multinational | 6.89 | -0.12 | 0.35 | -- | -- |  |  |  |  |
| Private national | 7.03 | 0.02 | 0.28 | 0.14 | 0.35 | -- | -- |  |  |
| Public | 6.87 | -0.14 | 0.30 | -0.02 | 0.36 | -0.16 | 0.30 | -- | -- |
| Highlands yellow |  |  |  |  |  |  |  |  |  |
| MasAgro | 6.62 | -- | -- |  |  |  |  |  |  |
| Multinational | 6.95 | 0.33 | 0.44 | -- | -- |  |  |  |  |
| Private national | 6.45 | -0.17 | 0.44 | -0.50 | 0.60 | -- | -- |  |  |
| Public | 5.81 | -0.81*** | 0.29 | -1.14** | 0.49 | -0.64 | 0.50 | -- | -- |
| Tropical white |  |  |  |  |  |  |  |  |  |
| MasAgro | 7.09 | -- | -- |  |  |  |  |  |  |
| Multinational | 6.87 | -0.22 | 0.28 | -- | -- |  |  |  |  |
| Private national | 6.45 | -0.64** | 0.27 | -0.42 | 0.33 | -- | -- |  |  |
| Public | 6.43 | -0.66*** | 0.25 | -0.43 | 0.31 | -0.01 | 0.30 | -- | -- |
| Tropical yellow |  |  |  |  |  |  |  |  |  |
| MasAgro | 6.41 | -- | -- |  |  |  |  |  |  |
| Multinational | 6.29 | -0.12 | 0.21 | -- | -- |  |  |  |  |
| Private national | 5.54 | -0.87*** | 0.20 | -0.75*** | 0.23 | -- | -- |  |  |
| Public | 6.08 | -0.33 | 0.41 | -0.21 | 0.43 | 0.54 | 0.42 | -- | -- |

Robust standard error *** p<0.01, ** p<0.05, * p<0.1, R-squared= 0.704, F-test= 45.91. Source: [33].
