# Supplementary material for: Public-private partnerships for seed industry development in developing countries: Lessons from MasAgro maize in Mexico
Source: PLoS One. 2025 Aug 6;20(8):e0328872. doi: 10.1371/journal.pone.0328872 (PMC12327655; doi:10.1371/journal.pone.0328872)
Supplement: S6 Fig — Source: [34,41,42]. (DOCX) [file pone.0328872.s008.docx]

**S6 Fig. Sampled MasAgro SMEs composition of maize seed sales by product type and company size, 2011-2019 (n=31).** Source: [34, 41, 42]. ^1^Refers to each category seed sales relative to the total sales of sampled companies.
